# miR‐203‐3p promotes senescence of mouse bone marrow mesenchymal stem cells via downregulation of Pbk

**DOI:** 10.1111/acel.14293

**Published:** 2024-08-09

**Authors:** Qiaojuan Mei, Kexin Li, Tianyu Tang, Siying Cai, Yu Liu, Xiaofei Wang, Yinzhao Jia, Ling Zhang, Huaibiao Li, Hui Song, Jun Zhai, Wenpei Xiang

**Affiliations:** ^1^ Institute of Reproductive Health, Tongji Medical College Huazhong University of Science and Technology Wuhan China; ^2^ Department of Gynecology Maternal and Child Health Hospital of Hubei Province Wuhan China; ^3^ The First College of Clinical Medical Science China Three Gorges University Yichang China; ^4^ Union Hospital, Tongji Medical College Huazhong University of Science and Technology Wuhan China; ^5^ Department of Cardiology, Jinan Central Hospital Shandong First Medical University Jinan Shandong China; ^6^ Center for Reproductive Medicine The First Affiliated Hospital of Zhengzhou University Zhengzhou China

**Keywords:** aging, bone marrow mesenchymal stem cells, miR‐203‐3p, osteoporosis, PDZ‐binding kinase

## Abstract

The senescence of bone marrow mesenchymal stem cells (BMSCs) contributes to the development of degenerative skeletal conditions. To date, the molecular mechanism resulting in BMSC senescence has not been fully understood. In this study, we identified a small non‐coding RNA, miR‐203‐3p, the expression of which was elevated in BMSCs from aged mice. On the other hand, overexpression of miR‐203‐3p in BMSCs from young mice reduced cell growth and enhanced their senescence. Mechanistically, PDZ‐linked kinase (PBK) is predicted to be the target of miR‐203‐3p. The binding of miR‐203‐3p to Pbk mRNA could decrease its expression, which in turn inhibited the ubiquitination‐mediated degradation of p53. Furthermore, the intravitreal injection of miR‐203‐3p‐inhibitor into the bone marrow cavity of aged mice attenuated BMSC senescence and osteoporosis in aged mice. Collectively, these findings suggest that targeting miR‐203‐3p to delay BMSC senescence could be a potential therapeutic strategy to alleviate age‐related osteoporosis.

## Graphical abstract

This study indicates that inhibiting miR‐203‐3p (partly via the PBK/p53 signaling pathway) improves cell growth dynamics and slows the process of cellular senescence, rejuvenating senescent BMSCs. Furthermore, it presents a new potential target for delaying age‐related osteoporosis.

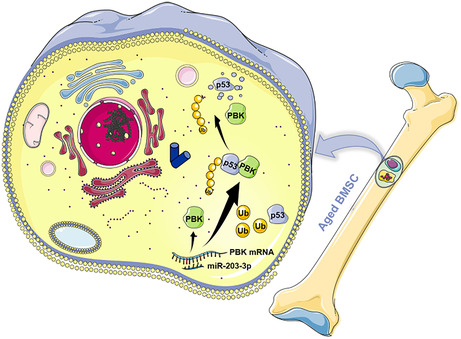

**Abbreviations**
AAV: adeno‐associated virus
ALP: alkaline phosphatase
BMSCs: bone marrow mesenchymal stem cells
BV/TV%: bone bulk density
DEGs: differential expression genes
FACS: Flow cytometry analysis
GO: gene ontology
IBMI: intra‐bone marrow injection
KEGG: kyoto encyclopedia of genes and genomes
micro‐CT: micro computed tomography
mtDNA: mitochondrial DNA
p16: cyclin dependent kinase inhibitor 2A
p21: cyclin dependent kinase inhibitor 1A
p53: transformation related protein 53
PBK: PDZ‐linked kinase
ROS: reactive oxygen species
ROS: reactive oxygen species
SASP: Senescence‐associated secretory phenotype
Tb.N: trabecular number
Tb.Th: trabecular thickness
Δψ m: mitochondrial membrane potential

## 1 INTRODUCTION

In broad terms, aging refers to the functional decline that occurs in most organisms with age (López‐Otín et al., 2013). This functional decline is proposed to be attributed to the dysfunction of tissue stem cells (Clevers & Watt, 2018). One of the causes for aging‐related functional decline is stem cell senescence (Liu et al., 2015). Aging MSCs have limited capacity of proliferation and differentiation, consequently unable to repair damaged tissue (Deng et al., 2019). In this regard, in order to develop interventions and treatments for degenerative diseases, it is essential to elucidate the underlying molecular mechanisms responsible for age‐related MSC dysfunction (Li et al., 2021).

Mesenchymal stem cells derived from the bone marrow (BMSCs) are extensively used in regenerative medicine and tissue engineering (Liu et al., 2021). It is increasingly established that the pluripotency and proliferation capacity of BMSCs decline with age, thus restricting their effectiveness in repairing damaged organs (Hu et al., 2022). The main characteristics of aging BMSCs include proliferation cessation, reduced self‐renewal, and impaired differentiation, leading to “stem cell depletion” in vivo (Baker et al., 2015), which further leads to impaired bone mass and delayed repair of long bones (Li et al., 2017; Liu, Zhang, et al., 2020). Loss of osteogenic and lipogenic differentiation ability to maintain homeostasis further impairs the ability of tissue regeneration and exacerbates the degree of damage associated with age‐related bone degenerative diseases. (Ambrosi et al., 2021). Hence, the understanding of molecular mechanisms that regulate the aging process of BMSCs would facilitate the preserving “BMSC rejuvenation” and thereby delaying the progression of bone degenerative diseases.

MicroRNAs (miRNAs) are small non‐coding ribonucleic acids (19–22 nt) that selectively bind to the 3′‐untranslated region (3′‐UTR) of messenger ribonucleic acids (mRNAs) (Ambros, 2003; Bartel, 2004; Lai, 2003). They modulate gene expression by restraining translation or stimulating the degradation of target mRNAs (He & Hannon, 2004). Given their control over targets genes, it is believed that miRNAs play important roles in physiological processes, such as proliferation, differentiation and senescence of BMSCs (Heiler et al., 2016). For example, miR‐21 regulates osteogenic and lipogenic differentiation of BMSC by targeting PTEN (Zhou et al., 2021), miR‐155‐5p mediates MSC senescence by regulating the Cab39/AMPK signaling pathway (Hong et al., 2020).

In this study, we identified a family of miRNA, miR‐203‐3p, the level of which remarkably increased in BMSCs from aged mice, compared with cells from young mice. This led to the hypothesis that upregulation of miR‐203‐3p promotes BMSC aging. To test this, we either knocked down or overexpressed miR‐203‐3p in old and young BMSCs, followed by phenotypic characterization. We also explored the underlying molecular mechanisms causing BMSCs senescence by miR‐203‐3p. Moreover, we tested the efficacy of miR‐203‐3p inhibition on preventing osteoporosis.

## 2 RESULTS

### 2.1 Aged BMSCs exhibited increased level of cellular senescence

**FIGURE 1:**
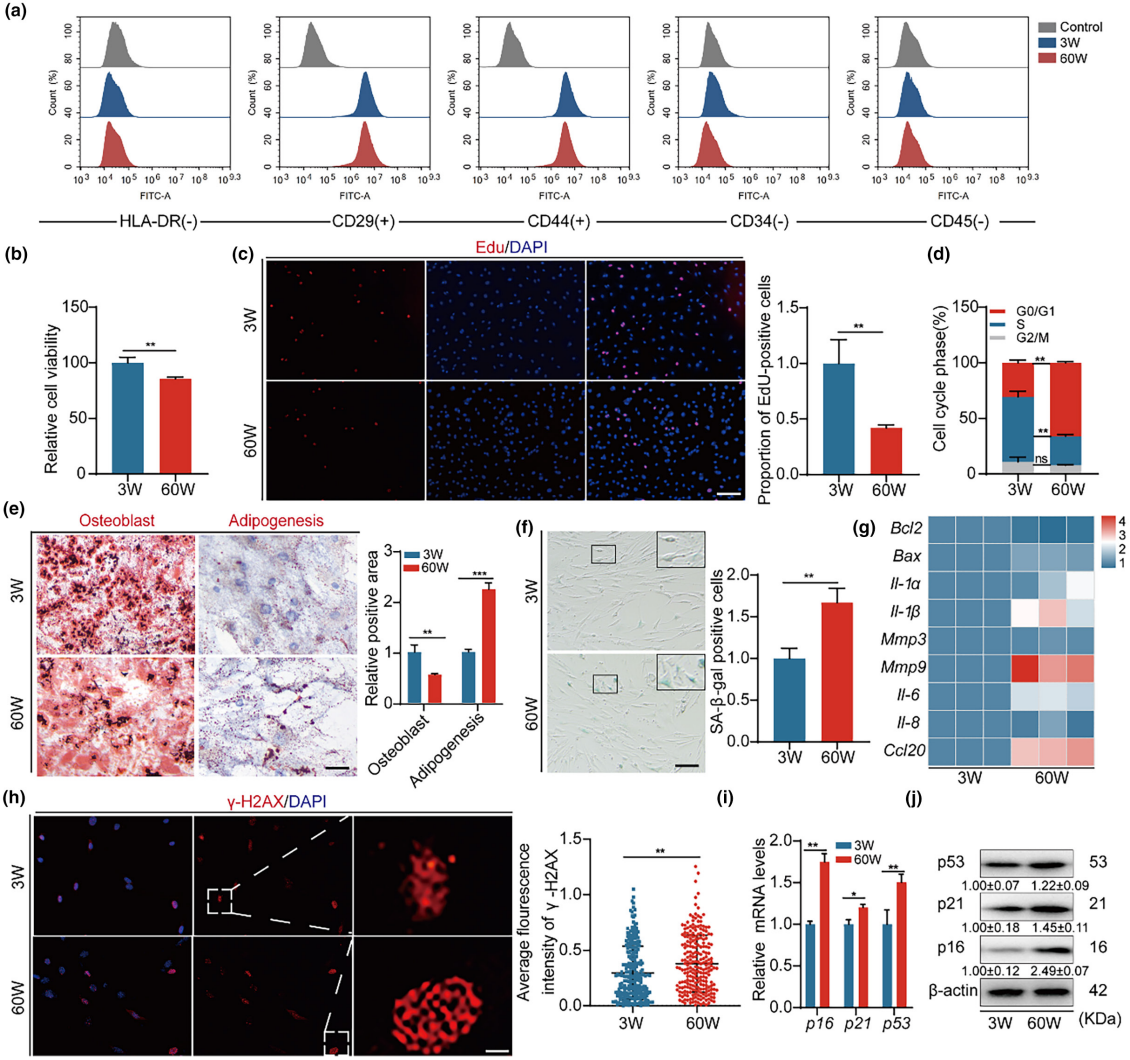
Aged BMSCs exhibited increased level of cellular senescence. (a) FACS analysis of BMSC surface markers CD29, CD44, CD34, CD45, and HLA‐DR. (b) CCK8 assay for cell viability. (c) Edu assay for cell proliferation and quantitative analysis of positive cells, Bar: 100 μm. (d) Flow cytometry for cell cycle of both cell groups and quantitative analysis of cycle distribution. (e) Both osteogenic differentiation and adipogenic differentiation in 3W and 60W BMSCs, determined by Alizarin red staining and Oil red staining with quantification of osteogenic and adipogenic efficiency, Bar: 200 μm. (f) SA‐β Gal assay to determine cellular senescence and quantitative analysis of positive cells, Bar: 100 μm. (g) Heatmap of mRNA expression of phenotype‐related genes for cellular senescence analysis. (h) γ‐H2ax immunofluorescence to detect cellular DNA damage and quantitative analysis of fluorescence intensity, Bar: 5 μm. (i, j) Cellular expression of p16, p21 and p53 mRNA and protein expression levels and quantitative analysis, β‐actin served as loading control. *p < 0.05; **p < 0.01; ***p < 0.001.

To evaluate the distinct characteristics of BMSCs from young and old mice, we isolated BMSCs from mice of 3 weeks (young BMSCs) and 60 (aged BMSCs) weeks old, respectively. Flow cytometry analysis showed that the isolated cells were positive for CD29 (99.7% vs. 99.4%) and CD44 (98.3% vs. 97.7%), surface markers of BMSCs, and negative for CD34 (1.9% vs. 1.7%) and CD45 (1.2% vs. 1.5%) (Figure 1a). We next assessed the growth and senescence of these cells. Compared with young BMSCs, aged BMSCs had lower level of viability and proliferation (Figure 1b,c). Flow cytometry quantification showed that majority of aged BMSCs were blocked in G1 phase (Figure 1d). At the same time, osteogenic and adipogenic differentiation were carried out, and aged BMSCs showed significantly reduced osteogenic differentiation potential but improved adipogenic differentiation, suggesting that the differentiation of osteogenesis and adipogenesis of aged BMSCs is unbalanced (Figure 1e). Furthermore, aged BMSCs exhibited a large proportion of SA‐β‐gal‐positive cells (Figure 1f), enhanced secretion of senescence‐associated factors (Figure 1g), and increased level of γ‐H2AX (Figure 1h). Additionally, the expression of senescence‐related genes, including p16, p21 and p53, dramatically increased in aged BMSCs (Figure 1i,j).

Since mitochondrial dysfunction is one of the features of BMSC aging (Zhang et al., 2018), we examined the morphology and function of mitochondria in young and aged BMSCs. We found that mitochondria in aged BMSC were disorganized, with reduced proportion of long rods (Figure S1a,b). The mitochondrial copy number (mtDNA), mitochondrial membrane potential (Δψ m) and ATP content and significantly decreased in aged BMSCs (Figure S1c–f). Of note, the level of reactive oxygen species (ROS) measured by DCFH‐DA probe and FACS measurement was significantly increased in aged BMSCs, indicating an impaired redox state in aged BMSCs (Figure S1g–i). Together, these findings suggest that there is an increase in the cellular senescence of aged BMSC.

### 2.2 miRNA sequencing revealed differential expression of miRNAs between 3W‐BMSC and 60W‐BMSC

To identify the differentially expressed miRNAs during BMSC aging, we next performed high‐throughput sequencing of the small non‐coding RNA transcriptomes of young and aged BMSCs. The heatmap displays miRNAs, the level of which differed significantly between the two groups (Figure S2a). A total of 952 miRNAs were identified and analyzed, out of which 32 were found to differentially express between young and old BMSCs (|log2(fold change)| >1 and adjusted *p* < 0.05 as cutoffs). Among these miRNAs, 17 miRNAs were upregulated, while 15 downregulated (Figure S2b). Enrichment analysis showed that pathways targeted by identified miRNAs include Ras signaling pathway, Wnt signaling pathway, and MAPK signaling pathway (Figure S2c,d).

To identify potentially significant miRNAs connected to BMSC senescence, we analyzed 32 differentially expressed genes from the sequencing results and compared them with 41 differentially expressed genes associated with the median lifespan of mice (Lee et al., 2017). This allowed us to identify two plausible target miRNAs, miR‐203‐3p and miR‐592‐5p (Figure S2e). Validated by a large sample, miR‐203‐3p was found to be more highly expressed in BMSCs (Figure S2f) and was significantly highly expressed in senescent BMSCs (1.91 ± 0.37). Thus, we postulate that miR‐203‐3p may have a vital role in the age‐increasing aging process in BMSC.

### 2.3 High expression of miR‐203‐3p in BMSCs leads to reduced cell growth and promotes senescence

**FIGURE 2:**
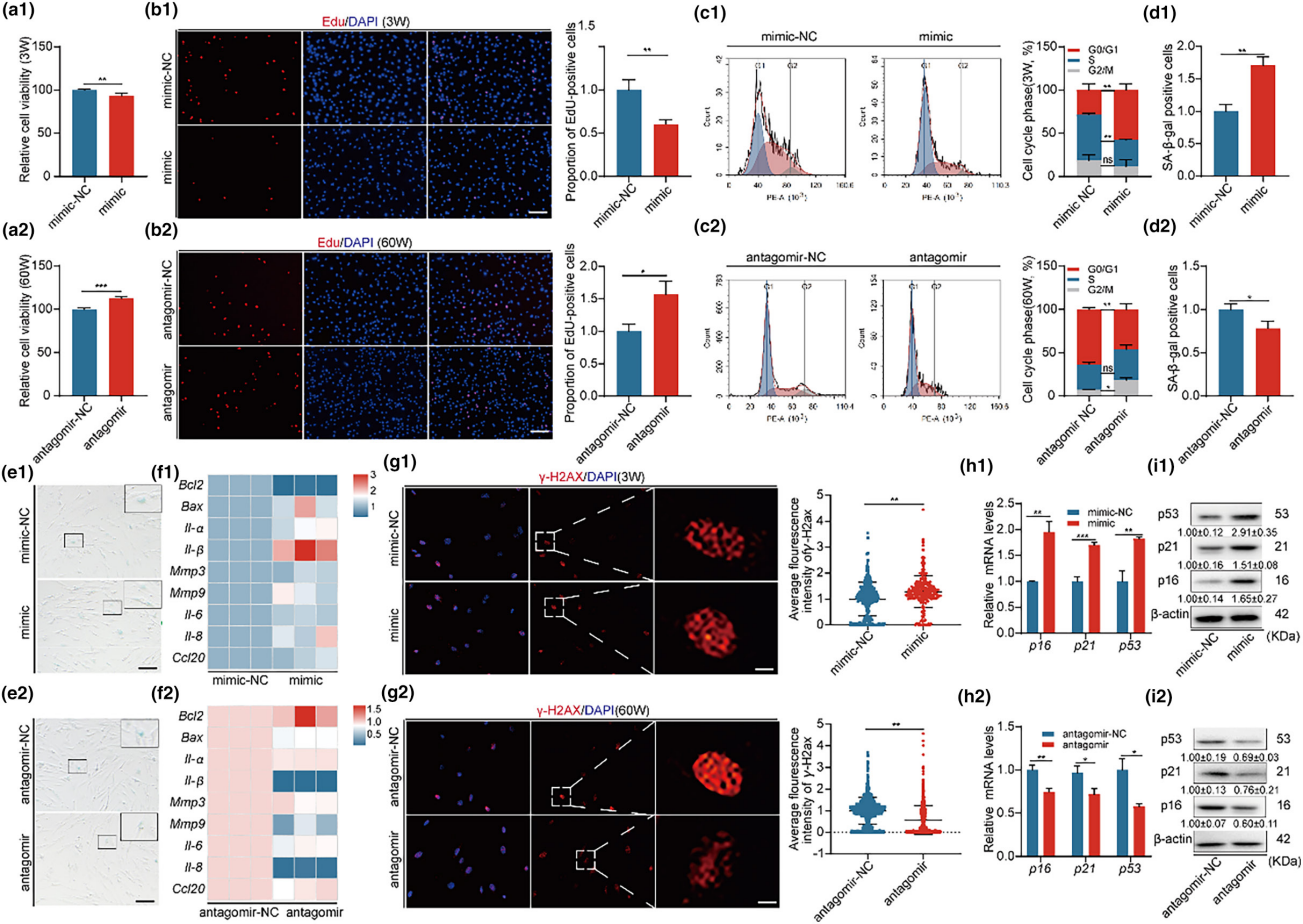
High expression of miR‐203‐3p in BMSCs leads to reduced cell growth and promotes senescence. Overexpression of miR‐203‐3p in 3W‐BMSCs and silencing of miR‐203‐3p in 60W‐BMSCs were assayed in the corresponding two groups of cells, respectively. (a1, a2) CCK8 assay for cell viability. (b1, b2) Edu assay for cell proliferation and quantitative analysis of positive cells, Bar: 100 μm. (c1, c2) Flow cytometry was performed to detect the cell cycle of both groups of cells and to quantify the cycle distribution. (d1, d2, e1, e2) SA‐β‐gal assay to determine cellular senescence and quantify positive cells, Bar: 100 μm. (f1, f2) Heatmap of mRNA expression of genes related to cellular senescence analysis phenotype. (g1, g2) γ‐H2ax immunofluorescence assay to detect cellular DNA damage and quantify the fluorescence intensity, Bar: 5 μm. (h1, h2, i1, i2) Expression levels and quantitative analysis of p16, p21, p53 mRNA, and protein in both groups of cells. β‐Actin was used as a loading control. *p < 0.05; **p < 0.01; ***p < 0.001.

To pinpoint the role of miR‐203‐3p in BMSC aging, we conducted loss and gain of function experiments. MiR‐203‐3p mimics were transfected into young BMSCs. Compared to the control, expression of miR‐203‐3p mimics resulted in decreased cell viability and proliferation of young BMSCs (Figure 2a1,b1), along with cell cycle arrest (Figure 2c1), while the osteogenic differentiation ability of the cells was weakened, and the lipogenic differentiation ability was enhanced (Figure S3a). Furthermore, an increased proportion of SA‐β‐gal‐positive cells as well as the level of senescence‐associated secretory phenotype (SASP) and γ‐H2AX was observed after miR‐203‐3p mimic transfection (Figure 2d–g). The levels of p16, p21, and p53 were also increased (Figure 2h1,i1), consistent with the phenotypes observed in aged BMSCs.

On the other hand, when aged BMSCs were treated with miR‐203‐3p inhibitor, cell growth was increased (Figure 2a2–c2) and the imbalance between osteogenic and lipogenic differentiation of the cells was reversed to some extent (Figure S3b). Furthermore, cellular senescence was rescued to a certain extent (Figure 2f2–i2). These results suggest that BMSC senescence is to some extent attributed to the upregulation of miR‐203‐3p.

### 2.4 Pbk is a target of miR‐203‐3p

**FIGURE 3:**
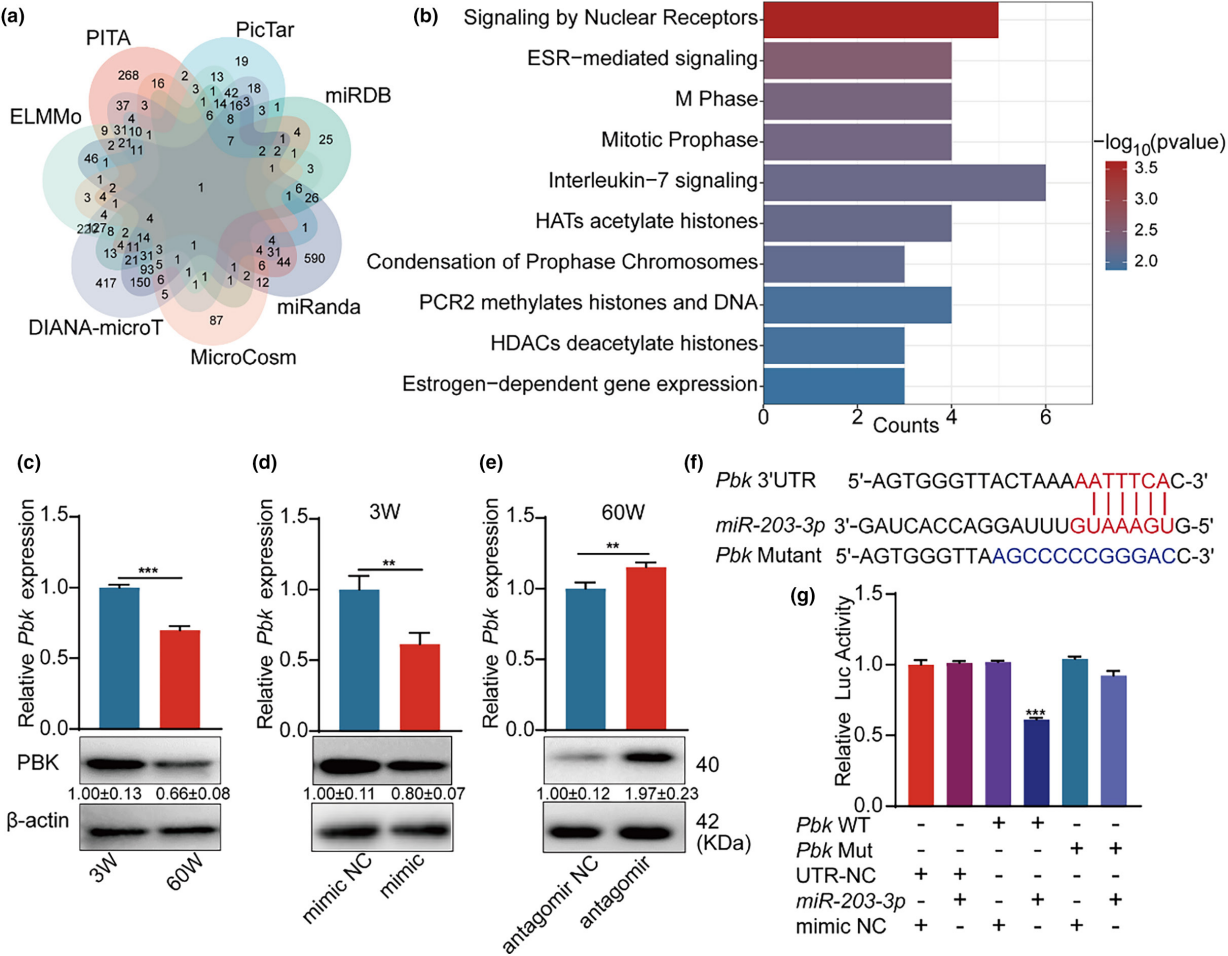
Pbk is a target of miR‐203‐3p. (a) Venn diagram of miR‐203‐3p target genes, overlapped according to seven predicted URLs (miRanda, MicroCosm, DIANA‐microT, ELMMo, PITA, PicTar and miRDB). (b) KEGG pathway of miR‐203‐3p target gene, mainly enriched in “M Phase, Mitotic Prophase.” (c) Relative expression of mRNA and protein of Pbk in young BMSCs and aged BMSCs as indicated. (d) Relative expression of mRNA and protein of the indicated Pbk after overexpression of miR‐203‐3p in young BMSCs. (e) Relative expression of mRNA and protein of the indicated Pbk after silencing of miR‐203‐3p in aged BMSCs. (f, g) Dual luciferase reporter gene analysis indicates that miR‐203‐3p can bind to the 3′‐UTR of Pbk. **p < 0.01; ***p < 0.001.

**FIGURE 4:**
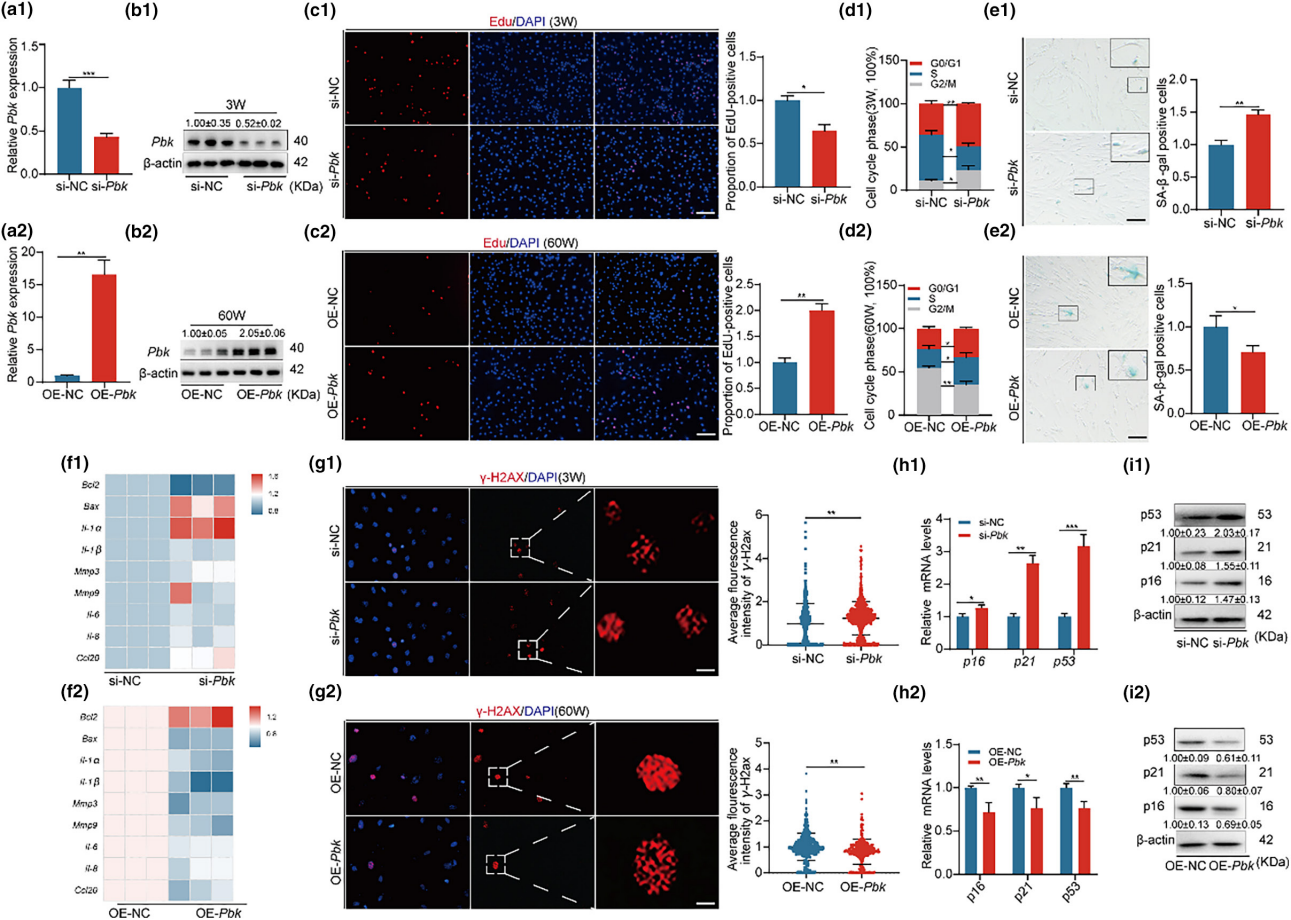
Pbk positively regulates the function of BMSC. (a1, a2, b1, b2) Pbk silencing in young BMSCs and Pbk overexpression in aged BMSCs. mRNA and protein expression levels were examined in the corresponding two groups of cells to confirm knockdown and overexpression efficiency, respectively. (c1, c2) Edu assay to detect cell proliferation and quantify positive cells, Bar: 100 μm. (d1, d2) Flow cytometry to detect cell cycle in both groups of cells and quantify cycle distribution. (e1, e2) SA‐β‐gal assay to determine cell senescence and quantify positive cells, Bar: 100 μm. (f1, f2) Heatmap of mRNA expression of phenotypically related genes for cell senescence analysis. (g1, g2) γ‐H2ax immunofluorescence to detect cellular DNA damage and quantify fluorescence intensity, Bar: 5 μm. (h1, h2, i1, i2) p16, p21, p53 mRNA and protein expression levels, and quantification in both groups of cells. β‐Actin was used as a loading control. *p < 0.05; **p < 0.01; ***p < 0.001.

To identify the mRNAs that miR‐203‐3p binds, we used seven miRNA target prediction tools (miRanda, MicroCosm, DIANA‐microT, ELMMo, PITA, PicTar, and miRDB) to predict its target (Figure 3a). We performed kyoto encyclopedia of genes and genomes (KEGG) signaling pathway enrichment with the predicted targets. M Phase and Mitotic Prophase pathways are within the top five enriched pathways (Figure 4b), suggesting that miR‐203‐3p regulates cell cycle‐associated genes. Among the predicted targets, we found 18 candidate miRNAs, as shown in Figure S4. Following verification via RT‐qPCR and Luciferase report, it was speculated that Pbk was a potential target for miR‐203‐3p in BMSCs, playing a major role in proliferation and in safeguarding mitotic fidelity in cells (Stauffer et al., 2017), which has been shown to be upregulated during mitosis and that Pbk binding and phosphorylation by CDK1/cyclin B is required for its mitotic activity (Abe et al., 2007). First, we analyzed the expression of Pbk in young and aged BMSCs. Pbk significantly decreased in aged BMSCs (0.66 ± 0.08), compared to young ones (Figure 4c). Next, we transfected miR‐203‐3p mimics into young BMSCs, which substantially decreased Pbk expression (0.80 ± 0.07) (Figure 4d). Nonetheless, transfection with miR‐203‐3p inhibitors raised the expression of Pbk in aged BMSCs (1.97 ± 0.23) (Figure 4e). These results imply that miR‐203‐3p suppresses the expression of Pbk in BMSCs.

TargetScan indicates that the 3′UTR of Pbk contains miR‐203‐3p binding site (Figure 4f). To determine whether Pbk is a direct target of miR‐203‐3p, the predicted miR‐203‐3p binding sequence within the 3′UTR was inserted into the downstream of the luciferase coding sequence. Luciferase reporter assays revealed that miR‐203‐3p inhibited the luciferase activity of the Pbk‐WT reporter. When the binding site was mutated, the inhibitory effect on Luciferase was rescued (0.61 ± 0.01) (Figure 4g), suggesting that miR‐203‐3p is capable of binding to Pbk mRNA directly, thus downregulating its level.

### 2.5 miR‐203‐3p promotes the senescence of BMSC by downregulating Pbk

Pbk, a crucial member of the MAPKK family of proteins that encodes serine/threonine protein kinases (Mukherjee et al., 2015), highly expresses in tumor cells and some stem cells (Kitamura et al., 2008). To examine the effect of Pbk on BMSC senescence, we conducted Pbk knockdown or overexpression in BMSCs. RT‐qPCR (Figure 4a1,b1) and Western blotting (Figure 4a2,b2) demonstrated the knockdown of Pbk (0.52 ± 0.02) and overexpression of Pbk (2.05 ± 0.06).

We then examined how the changed level of Pbk affects the growth of BMSCs. When Pbk was knocked down in young BMSCs, proliferation of these cells decreased, as shown by quantification of EdU‐positive cells (see Figure 4c1) and cell cycle analysis (Figure 4d1). Meanwhile, the balance of osteogenic and lipogenic differentiation of cells is disrupted (Figure S5a). We further observed the impact of Pbk downregulation on the senescence of young BMSCs. Knockdown of Pbk in young BMSCs led to an increase of SA‐β‐gal positive cells (Figure 4e1), elevated SASP (Figure 4f1), DNA breakpoints (Figure 4g1), and p16, p21, p53 mRNA and protein expression levels (Figure 4h1,i1). These results indicate that inhibiting Pbk promotes increased cellular senescence. On the hand, the overexpression of Pbk in aged BMSCs enhanced their proliferation (Figure 4c2,d2) and balance of differentiation of bone and lipid (Figure S5b). Moreover, upregulation of Pbk in aged cells rescued cellular senescence (Figure 4e2–i2).

We next sought to further explore whether Pbk could reverse miR‐203‐3p‐mediated cellular phenotypes. Upon overexpression of miR‐203‐3p in young BMSCs, we found that Pbk overexpression effectively reversed the miR‐203‐3p mimic‐induced reduction in cell viability (Figure S6a), proliferation capacity (Figure S6b–d) and the balance of osteoblastic, and lipogenic differentiation of cells was restored (Figure S6e,f). Also, senescence of cells transfected with miR‐203‐3p mimic was significantly alleviated. This was manifested as a reduction in the proportion of SASP (Figure S2g), SA‐β‐gal positive cells (Figure S2h,i), p16, p21, p53 protein levels (Figure S2j), and DNA breakpoints (Figure S2k,l).

Taken together, these findings indicate that downregulation of Pbk by miR‐203‐3p during aging promotes the senescence of BMSCs.

### 2.6 Pbk promotes the degradation of p53

**FIGURE 5:**
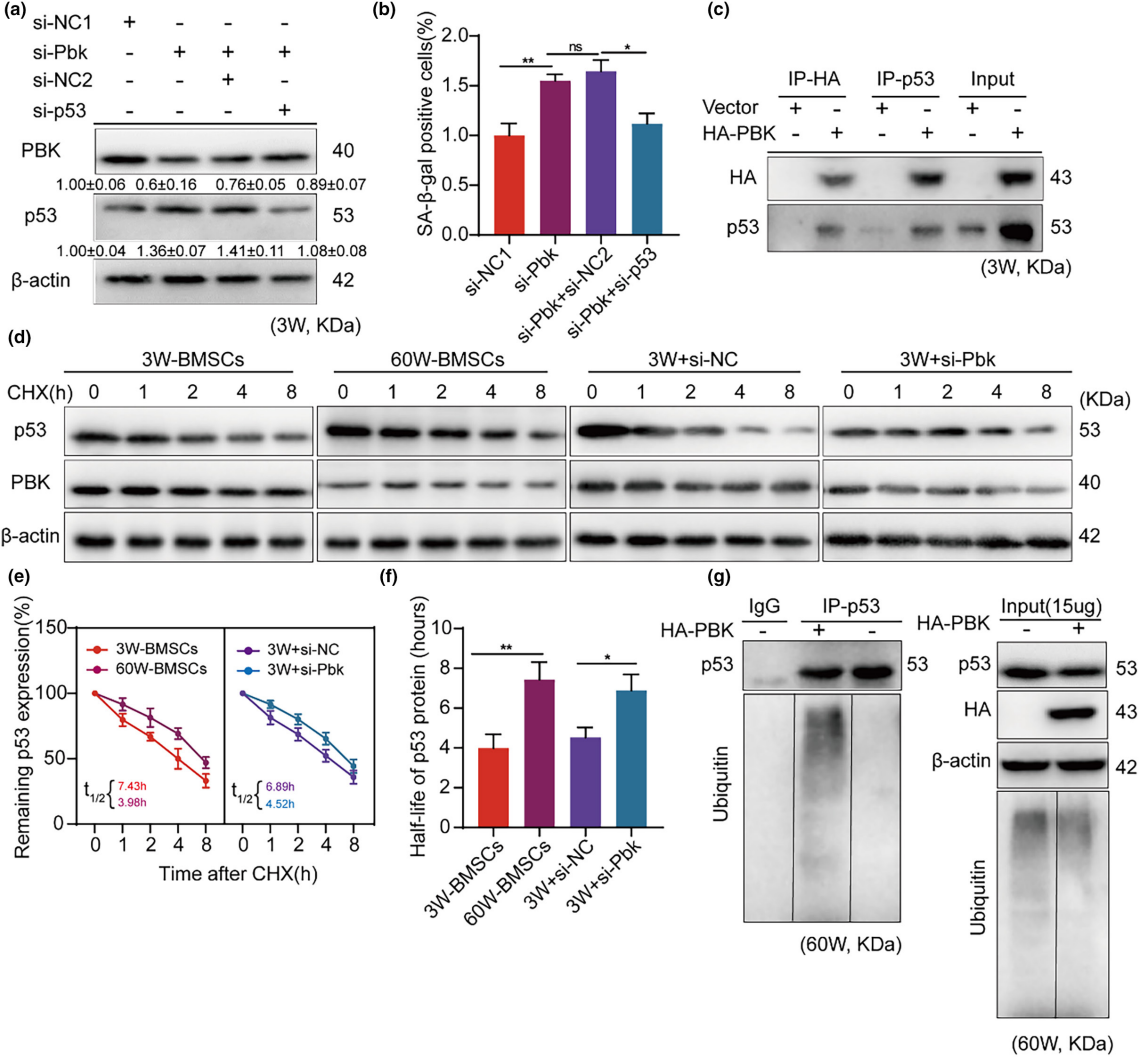
Pbk promotes the degradation of p53. (a) The expression of Pbk and p53 was determined by protein blotting in young BMSCs after knockdown of the Pbk group and subsequent p53 treatment. (b) The rates of SA‐β‐gal‐positive cells in the four groups were compared and statistically analysed after knockdown of the Pbk group and then p53 treatment in 3W‐BMSCs. (c) Immunoprecipitation analysis of HA‐pbk transfected 60W BMSCs by protein blotting with anti‐HA or anti‐p53 antibodies. (d–f) Cycloheximide treatment followed by protein blotting for p53 protein stability analysis. (g) Ubiquitination assay in BMSC with and without ectopic Pbk expression. Immunoprecipitation of control IgG and p53 and protein blotting using anti‐p53 and anti‐ubiquitin antibodies to detect ubiquitin proteins (left). Identical loading levels of proteins and global ubiquitination profiles are shown (right). β‐Actin was used as a loading control. *p < 0.05; **p < 0.01.

The p53 gene, a crucial tumor suppressor, restrains cell growth by inducing cell cycle arrest (Kong et al., 2019). Numerous studies have indicated that Pbk regulates p53 activity via direct or indirect mechanisms (Park et al., 2020). Thus, we inferred that Pbk inhibits senescence by downregulating p53. The mRNA and protein levels of p53 were analyzed using RT‐qPCR and Western blot in previous experiments. In aged BMSC, p53 expression was significantly high (1.22 ± 0.09) (Figure 1j). Similar downregulation was seen when miR‐203‐3p was overexpressed (2.91 ± 0.35) (Figure 2i1), or when Pbk was knocked down (2.03 ± 0.17) (Figure 4i1). Intriguingly, in 3W‐BMSC, knockdown of p53 partially mitigated the cellular senescence caused by Pbk knockdown (Figure 5a,b).

To shed light on how Pbk modulates p53 in BMSCs, we performed immunoprecipitation to isolate ectopically expressed Pbk or endogenous p53. The results showed that Pbk interacted with p53 (Figure 5c). To evaluate the post‐translational regulation of p53, we analysed protein stability of p53 by cycloheximide pulse‐chase analysis. The analysis showed that the stability of p53 protein was considerably increased in aged BMSC, compared with young BMSCs (3.99 ± 0.71 vs. 7.44 ± 0.87). This trend could be recapitulated in young BMSCs by knocking down Pbk (4.53 ± 0.52 vs.6.89 ± 0.84) (Figure 5d–f). As p53 is reported to be degraded by ubiquitination (Liu, Guan, et al., 2020). we investigated whether Pbk‐dependent p53 ubiquitination is present. The results showed that, though the global ubiquitination was not affected by Pbk overexpression in BMSCs, the level of ubiquitinated p53 was significantly increased (Figure 5g). These results imply that the suppression of Pbk by miR‐203‐3p could prevent the ubiquitin‐mediated degradation of p53, which in turn promotes the senescence of BMSCs.

### 2.7 Inhibition of miR‐203‐3p expression delays osteoporosis in aged mice

**FIGURE 6:**
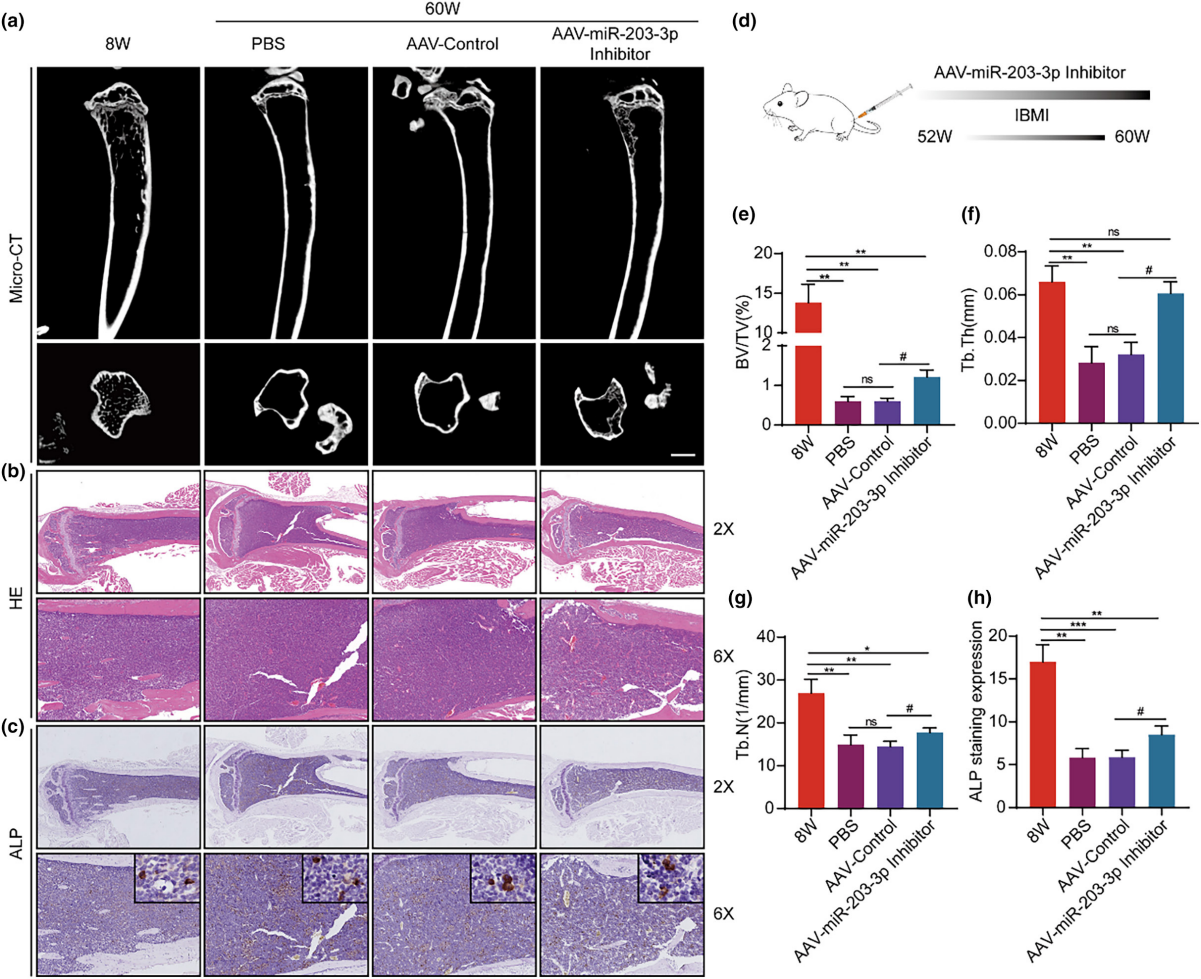
Inhibition of miR‐203‐3p expression delays osteoporosis in aged mice. (a, e, f, g) Representative micro‐CT coronal and sagittal views of the mouse fibula. CTAn was used to measure bone bulk density (BV/TV%), trabecular thickness (Tb.Th, mm) and trabecular number (Tb.N, mm^−1^). (b) Representative H&E‐stained sections of mouse fibula, Bar: 5 μm. (c, h) Representative ALP‐stained sections of mouse fibula and statistical evaluation of ALP staining expression. (d) Schematic representation of the process of miR‐203‐3p AAV inhibitor injection into the bone marrow cavity of mice. *p < 0.05; **p < 0.01; ***p < 0.001; ^#^ *p* < 0.05.

The results above showed that the inhibition of miR‐203‐3p helped postpone BMSC senescence. Consequently, we aimed to investigate if in vivo downregulation of miR‐203‐3p in senescent BMSC improves their function. We tested the efficacy of the viral transfection in vitro by means of RT‐qPCR and Western blot (Figure S7). To this end, we injected the adeno‐associated virus (AAV) containing the miR‐203‐3p‐inhibitor into the bone marrow cavity of C57 female mice of 52 weeks old (Figure 6d), 8 weeks after administration, mice injected with the inhibitor displayed significant improvement in the bone volume fraction (0.61 ± 0.08 vs. 1.2 ± 0.18) (Figure 6a,e), bone trabecular thickness (0.03 ± 0.006 vs. 0.06 ± 0.005) (Figure 6f), and trabecular number (14.5 ± 1.18 vs. 17.8 ± 1.06) (Figure 6g), compared to the control group. H&E and ALP staining (5.87 ± 0.81 vs. 8.63 ± 0.83) showed that the inhibition of miR‐203‐3p in the bone marrow lumen delayed the loss of osteoblasts (Figure 6b,c,h).

We reasoned that the observed outcome was due to the inhibition of miR‐203‐3p expression in senescent BMSCs in the bone marrow cavity. To test this, we examined the expression of Pbk and p53 in the bone marrow cavity after the administration of the inhibitor. The injection of miR‐203‐3p inhibitor resulted in the increase of Pbk (0.58 ± 0.05 vs. 0.73 ± 0.04) (Figure S8a,b) and the reduction of p53 (13.7 ± 2.22 vs. 8.03 ± 1.40) (Figure S8c,d) in the bone marrow cavity, compared to the control group. This suggests that miR‐203‐3p could be a potential target for developing strategies to ameliorate aging‐associated osteoporosis.

## 3 DISCUSSION

Stem cell exhaustion is a primary cause of the aging process in organisms (López‐Otín et al., 2023). Therefore, preserving stem cells homeostasis is a promising anti‐aging and disease prevention measure. Our findings indicate that BMSC samples from older mice exhibit decreased cell growth kinetics, reduced mitochondrial function, and increased cellular senescence. These results are consistent with previous reports, confirming that cellular function in BMSC diminishes as the organism ages (Wagner et al., 2009). Consequently, promoting the rejuvenation and health of BMSC is beneficial.

In recent years, with an in‐depth study of miRNAs, the role of miRNAs in regulating BMSC senescence has emerged gradually. Studies have found that miRNAs can regulate BMSC function and further modulate the skeletal microenvironment by affecting mitochondrial function (Liu et al., 2019), osteoblast/osteoclast differentiation (Tang et al., 2023), and cell proliferation (Liu et al., 2022). In 2017, a high‐throughput miRNA array analysis of the median mouse species revealed that miR‐203‐3p expression was significantly negatively correlated with longer lifespan (Lee et al., 2017). These findings suggest a potential link between its miRNAs and the onset of organismal aging. Further studies have shown that increased expression levels of miR‐203‐3p inhibit proliferation and induce senescence in human melanoma cells (Jackson et al., 2013; Lena et al., 2008). Studies have observed that this miRNA is upregulated in WI‐38 human diploid fibroblasts (Marasa et al., 2010) and in a model of senescence in human melanoma cells (Noguchi et al., 2012). In line with these previous studies, the present study observed a significant increase in miR‐203‐3p in aged BMSCs, indicating that miR‐203‐3p could act as a potential factor in regulating MSC senescence. Moreover, our findings showed that upregulation of miR‐203‐3p in young BMSCs enhanced the cellular senescence phenotype, resulting in increased SA‐β‐gal positivity, SASP, DNA breakpoint, p16, p21, and p53 expression. Additionally, the cell growth kinetics of miR‐203‐3p mimic‐treated young BMSCs were downregulated. Conversely, suppressing miR‐203‐3p in BMSC from aged mice reduced cellular senescence while promoting cell proliferation. Injecting AAV (AAV‐miR‐203‐3p‐inhibitor) containing the miR‐203‐3p‐inhibitor plasmid into the bone marrow cavity of old mice was also successful in postponing the development of osteoporosis. These findings verify that the overexpression of miR‐203‐3p speeds up MSC senescence, whereas inhibiting miR‐203‐3p reinvigorates senescent BMSCs and safeguards against skeletal degenerative diseases. Nevertheless, the precise mechanism by which miR‐203‐3p regulates BMSC senescence still requires further investigation.

Cellular senescence is a stress response that prevents abnormal cell proliferation by inducing irreversible cell cycle arrest (Campisi & d'Adda di Fagagna, 2007). Repairing damages to respond to cellular stress and sustaining a normal cell cycle are crucial to delay cellular senescence. We coincidentally observed that predicted target genes of miR‐203‐3p were significantly enriched in cell cycle‐related signaling pathways. Consequently, we focused on whether miR‐203‐3p was associated with this classic regulated pathway, and we finally identified Pbk as the target of miR‐203‐3p. We determined that the expression of Pbk was considerably diminished in aged BMSCs. Moreover, overexpressing Pbk at young BMSCs effectively recovered the reduced proliferation of cells caused by the miR‐203‐3p mimic, which led to cellular senescence. The obtained result emphasizes and reinforces the role of Pbk as a highly conserved multifunctional protein in repairing cellular senescence damage and regulating cell cycle progression (Yu et al., 2008). Prior researches have indicated that Pbk blocks p53 expression or function by attaching itself to the DNA‐binding domain of p53 (Elson et al., 2000; Park et al., 2020). Our study also found comparable Pbk and p53 interactions in BMSCs, with greater p53 transcriptional activity in aged BMSCs, which was also noticed in Pbk knockdown cells. Furthermore, increasing Pbk overexpression did not have an effect on global ubiquitination in BMSC, but the level of ubiquitination conjoined with p53 was significantly raised, indicating that Pbk causes p53 to undergo ubiquitination, which then promotes degradation and plays its part in regulating BMSC's cell cycle and senescence. These discoveries offer new proof for Pbk‐monitored regulation of p53 expression.

This study has several limitations. Firstly, this study only investigated miR‐203‐3p in BMSC from aged mice, and the functions of other significantly‐enriched miRNAs require further investigation. Secondly, it has not been investigated if miR‐203‐3p regulates other targets besides Pbk to mediate senescence in MSCs. Finally, this study didn't obtain direct evidence of inhibiting miR‐203‐3 expression in senescent BMSCs to delay osteoporosis in the bone marrow cavity. In future studies, more robust and unbiased data, such as phenotypic observations of miR‐203‐3p‐KO mice and multi‐omics‐based screening, are needed to validate the findings.

In conclusion, our study indicates that inhibiting miR‐203‐3p (partly via the PBK/p53 signaling pathway) improves cell growth dynamics and slows the process of cellular senescence, rejuvenating senescent MSCs. Furthermore, it presents a new potential target for delaying age‐related osteoporosis.

## 4 MATERIALS AND METHODS

### 4.1 Experimental animals and study approval

Aging female C57BL/6J mice (>60 weeks, SPF class) and young female mice (3 weeks, SPF class) were obtained from the Animal Research Center of Huazhong University of Science and Technology. All mice were adapted for 7 days after their purchase and were maintained under a controlled temperature (26 ± 2°C) with a 12 h light/dark conditions. All animal care protocols and experiments were reviewed and approved by the Animal Care and Use Committees of the Laboratory Animal Research Center at Tongji Medical College, Huazhong University of Science and Technology, and this study was compliant with all relevant ethical regulations regarding animal research.

### 4.2 Primary cell culture and identification

The primary culture of BMSCs was performed as described previously (Hong et al., 2020; Xu et al., 2018). The primary BMSC were isolated and purified from aging female mice (60 weeks) and young female mice (3 weeks). After the sterile extraction operation of femurs and tibiae, the samples were washed twice in PBS (10010049, Gibco, USA). The bone marrow was repeatedly flushed out with complete medium that contained a‐MEM (M0200, Gibco, USA) basal medium, 10% (v/v) FBS (10099141, Gibco, USA) and 100 U/mL penicillin‐100 mg/mL streptomycin (10378016, Gibco, USA), centrifuged at 1000*g* for 3 min, resuspended in a‐MEM complete medium, and cultured in a humidified cell culture incubator (37°C, 5% CO_2_), cells could be passaged when they grew to more than 80% confluence. Adherent cells in passage 2 (P2) identified by flow cytometry were characterized by the existence of the typical surface antigens CD29 (11029180, eBioscience, USA) and CD44 (11044181, eBioscience, USA), as well as the absence of the hematopoietic markers CD34 (11034181, eBioscience, USA) and CD45 (11045181, eBioscience, USA). BMSC from P2 to P5 were used for experiment.

### 4.3 Cell viability and proliferation assays

Cell viability was determined using the Cell Counting Kit‐8 (C0037, Beyotime, China). After cells treatment, 10 μL/well CCK‐8 solution was added to each well of the 96‐well plate and incubated at 37°C for 1 h. The spectrophotometric absorbance increase at 450 nm was measured with a Synergy HTX Multi‐Mode Reader (BioTek, USA).

Cell proliferation capacity was analyzed with Cell‐Light EdU Apollo567 In Vitro Kit (C10338, RiboBio, China). Cells were exposed to 50 μM EdU for 2 h at 37°C and by fixed in 4% paraformaldehyde. Cells were then permeabilized using 0.5% Triton‐X‐100 and then reacted with Apollo488 for 30 min. Subsequently, Hoechst 33342 was used to stain the DNA contents of the cells for 30 min.

Cell cycle phase was performed using the Cell Cycle Assay Kit (C1052, Beyotime, China). Cells were digested with trypsin and subsequently fixed with 70% ethanol at 4°C for 12 h. The fixed cells were then stained with PI solution at 37°C for 30 min, all solutions used were prepared according to the instructions, and data was acquired using NovoCyte Flow Cytometer Systems (Agilent, USA).

### 4.4 Osteogenic and lipogenic differentiation of BMSCs

Mouse BMSCs osteogenic differentiation basal medium (PD‐003, Pricella, China) and mouse BMSCs lipogenic differentiation basal medium (PD‐004, Pricella, China) were used. BMSCs in all groups were differentiated into bone and lipid, and cultured for 14 days. Alizarin red staining evaluated bone formation from bone marrow mesenchymal stem cells, and oil red O staining evaluated lipid formation from bone marrow mesenchymal stem cells.

### 4.5 SA‐β‐gal staining

SA‐β‐gal staining was performed using the Cellular Senescence β‐Galactosidase Staining Kit (C0602, Beyotime, China). Cells were fixed at room temperature for 15 min at the end of culture using fixative solution, and then stained overnight at 37°C by adding staining working solution, all solutions used were prepared according to the instructions.

### 4.6 Immunofluorescence staining

Cells were fixed with 4% paraformaldehyde and permeated with 0.5% Triton X‐100 in PBS, then blocked with goat serum and incubated with indicated primary antibodies and secondary antibodies. For nuclear staining, the cells and sections were incubated with fluorescent dye DAPI staining reagent (G1012, Servicebio, China). Fluorescence imaging was performed using a Zeiss Axio Observer 5 fluorescence microscope (Carl Zeiss). Antibodies used are indicated in Table S1.

### 4.7 Real‐time fluorescence quantitative PCR (RT‐qPCR)

Total RNA was extracted from cultured cells using RNA‐easy (R701‐01, Vazyme, China), and then the reverse transcription reaction of the extracted RNA was performed using the All‐in‐One™ miRNA First‐Strand cDNA Synthesis Kit (4208C, GeneCopoeia, USA). To clarify the relative mRNA expression of the target genes, the All‐in‐One™ miRNA qPCR Primer (D0101A, GeneCopoeia, USA) was used for amplification reactions and the Quantagene q225 real‐time PCR system (Kubo, China) was used to collect real‐time fluorescence equivalents. The relevant primers used for RT‐qPCR are shown in Table S2.

### 4.8 Western blot

Total protein was obtained from the cultured cells using RIPA (P0013B, Beyotime, China) and protease inhibitor mixture (P1010, Beyotime, China), followed by electrophoresis using 15% SDS‐PAGE gel (G2003, Servicebio, China) and protein transfer on Polyvinylidene fluoride (PVDF) immunoblotting membrane (3010040001, Roche, USA). Antibody dilution ratios were configured according to the instructions. In this experiment, the primary antibody dilution ratio was 1:1000, and the secondary antibody was selected according to the primary antibody species, and the dilution ratio was 1:5000–1:10,000. Finally, the bands were developed by chemiluminescence using the electrochemiluminescence (ECL) reagent kit (SQ101, EpiZyme, China) and the relative expression of the target protein was calculated. Antibodies used are indicated in Table S1.

### 4.9 Mitochondrial function‐related assays

Intracellular ATP levels were measured using the ATP Assay Kit (S0026, Beyotime, China). Briefly, 100 μL of ATP assay working solution was added to the assay wells and 20 μL of sample was added after 3–5 min at room temperature, and the relative light unit (RLU) value was measured with a Synergy HTX Multi‐Mode Reader (BioTek). all solutions used were prepared according to the instructions. In addition, the cell membrane potential assay was performed by Enhanced mitochondrial membrane potential assay kit with JC‐1 (C1049B, Beyotime, China); the mitochondrial markers of living cells were stained by Mito‐Tracker Red CMXRos (C2003S, Beyotime, China); the mtDNA was detected by Tianamp genomic DNA kit (DP304, TIANGEN, China), and the cell DNA was amplified by specific mitochondrial DNA primers, the primer sequences are shown in Table S2.

### 4.10 miRNA‐sequencing

The miRNA sequencing samples involved in the experiment were BMSCs isolated from 3 W and 60 W C57 female mice, which were cultured in vitro to P2 generation for surface antigen identification. After qualified identification, miRNA sequencing samples were sent, and samples were repeated three times in each group. Total RNA was extracted from 3 W‐BMSC and 60‐BMSC using Trizol reagent (15596‐018, Invitrogen, USA), and the quality and quantity of RNA samples were assessed using a NanoDrop 2000 spectrophotometer (Thermo Fisher, USA). Library construction and miRNA sequencing were performed by Crystal Energy Biotechnology (Shanghai, China), which used the illumina Hiseq sequencing platform in single‐end 50 bp sequencing mode for high‐throughput sequencing of the samples, and used fastx_clipper for quality control of the raw data, and compared the obtained reads of each sample to the existing miRNA database (miRBase) and the results of new miRNA prediction to calculate the relative expression of miRNAs.

### 4.11 Dual luciferase reporter assay

Luciferase activity assays were performed in transiently transfected HEK293 cells. Samples were added to the measurement tube in a volume of 20 μL and an additional 20 μL Firefly Luciferase Assay Reagent was added and mixed 2–3 times. Firefly and Renilla luciferase activities were measured by a dual glo luciferase assay system (Promega, USA). Specific information is provided in Supplementary material 1.

### 4.12 Micro‐CT scanning

The mouse femur was fixed and then scanned by Micro‐CT with a tube current of 200 uA and a voltage of 70 KV, scanning the entire object. Then the original images were reconstructed using the 3D reconstruction software NRecon (software version V1.7.4.2, Bruker, Germany) for selected regions, and the ROI of the region of interest was analyzed using CT Analyser (software version 1.20.3.0, Bruker, Germany).

### 4.13 BCIP/NBP ALP staining

Paraffin‐embedded tissue sections were deparaffinized with xylene and rehydrated with an alcohol gradient and water. Staining was performed using the BCIP/NBT alkaline phosphatase chromogenic kit (C3206, Beyotime, China), and image capture and analysis were performed using an ortho‐fluorescence microscope (Carl Zeiss, Germany) after termination of the chromogenic reaction.

### 4.14 Protein stability assay

To measure protein stability, cells were treated with cycloheximide (CHX, HY‐12320, MCE, USA) after corresponding transfection, and proteins were obtained after the corresponding time of treatment, followed by Western blot to detect the expression of P53 relative to Pbk.

### 4.15 Co‐immunoprecipitation and in vivo ubiquitination assay

To investigate the interaction of the two proteins, cells were treated with the relative treatments and then immunoprecipitated using the immunoprecipitation (IP/CoIP) kit (abs955, absin, China) was used to detect antigen–antibody binding with anti‐P53 and anti‐HA monoclonal antibodies, respectively, to probe protein interactions. To further explore the level of intracellular ubiquitination, proteins were separated by SDS‐gel electrophoresis using immunoprecipitated proteins and detected by immunoblotting with anti‐P53 and anti‐ubiquitin monoclonal antibodies. Antibodies used are indicated in Table S1.

### 4.16 Adeno‐associated virus bone marrow injection

To verify whether inhibition of miR‐203‐3p can save dry senescence in vivo, we packaged mmu‐miR‐203‐3p Inhibitor AAV. Finally, the titer of mmu‐miR‐203‐3p Inhibitor AAV was 1.12E+13 VG/mL, and the dose of each side marrow cavity of mice was 10 μL. The drug was administered to C57 female mice at 52 weeks, and the relevant test was performed after 8 weeks of drug action. For detailed information, see Supplementary materials 2 and 3.

### 4.17 Statistical analysis

All data are presented as means ± SEM. Statistical analysis was performed by unpaired two‐tailed Student's *t* test, or one‐way or two‐way analysis of variance (ANOVA) followed by Bonferroni's post‐test, in Prism GrarhPad 8.0 (version 8.0c, GraphPad Software, Inc, USA). *p* < 0.05 was statistically significant.

## AUTHOR CONTRIBUTIONS

Wenpei Xiang, Jun Zhai and Hui Song designed the study and published this manuscript; Qiaojuan Mei and Kexin Li performed the experiments and wrote the manuscript; Yu Liu, Xiaofei Wang, and Yinzhao Jia analyzed the data and collected the samples; Ling Zhang and Huaibiao Li provided their invaluable contributions in critically revising the manuscript.

## CONFLICT OF INTEREST STATEMENT

The authors declare that they have no known competing financial interests or personal relationships that could have appeared to influence the work reported in this paper.

## Supporting information


**Data S1:** Supporting Information.

## Data Availability

All data associated with this study are present in the paper or the Supplementary Materials except for RNA‐seq data. Requests for RNA‐seq data generated in this study should be sent to Wenpei Xiang.
