# Supplementary material for: miR‐203‐3p promotes senescence of mouse bone marrow mesenchymal stem cells via downregulation of Pbk
Source: Aging Cell. 2024 Aug 9;23(11):e14293. doi: 10.1111/acel.14293 (PMC11561657; doi:10.1111/acel.14293)
Supplement: Supplementary file 1 — Data S1: Supporting Information. [file ACEL-23-e14293-s001.docx]

**This supporting information includes:**

**FigureS1. Aged BMSCs have significantly reduced mitochondrial function**

**FigureS2. miRNA sequencing revealed differential expression of miRNAs between 3W-BMSC and 60W-BMSC**

**FigureS3. Effects of miR-203-3p on osteogenic and lipogenic differentiation of BMSCs**

**FigureS4. Screening of miR-203-3p target genes**

**FigureS5. Effect of Pbk on osteogenic and lipogenic differentiation of BMSCs**

**FigureS6. miR-203-3p promotes the senescence of BMSC by downregulating Pbk**

**FigureS7. miR-203-3p Inhibitor adeno-associated virus efficiency verification**

**FigureS8. Expression of Pbk/p53 in the bone marrow cavity of mice**

**Supplementary Table 1. Antibodies**

**Supplementary table 2. Primer sequences used in this study.**

**Supplementary material 1: Dual luciferase reporter assay**

**Supplementary material 2: mmu-miR-203-3p Inhibitor adeno-associated virus**

**Supplementary material 3: Packaging of mmu-miR-203-3p Inhibitor adeno-associated virus**


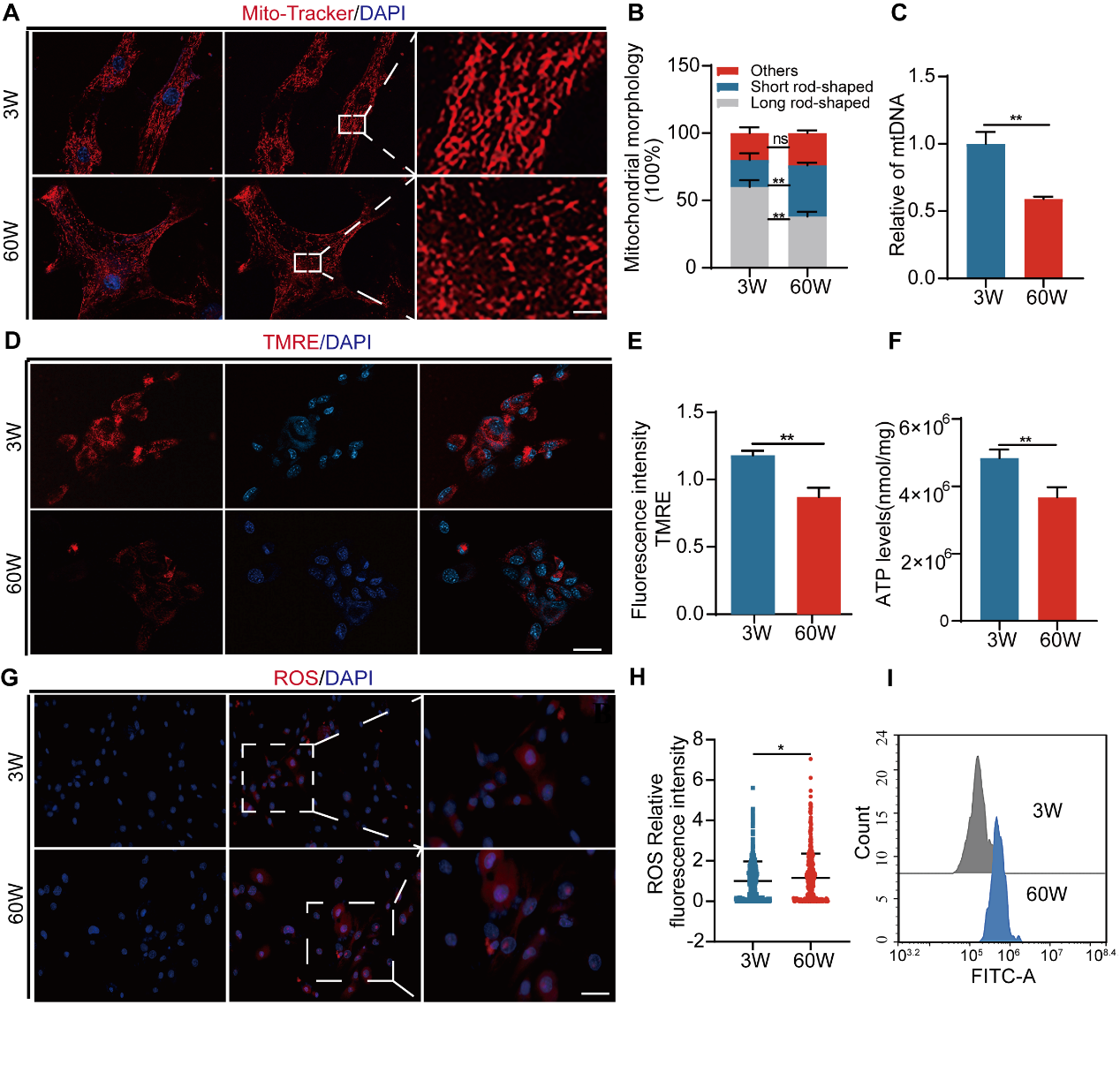


**FigureS1. Aged BMSCs have significantly reduced mitochondrial function**

**(A-B)** Mitochondria were stained with MitoTracker (red) and mitochondrial morphology (long rod, short rod, other) was quantified, Bar: 100 μm. **(C)** Relative expression of mtDNA in the two groups of cells. **(D-E)** Mitochondrial membrane potential was detected and quantified using the TMRE assay, Bar: 200 μm. **(F)** Measurement of ATP content in both groups of cells. **(G-I)** Cellular ROS content was detected using the DCFH-DA probe and FACS measurements, the fluorescence intensity was quantified, Bar: 200 μm. *, *P* < 0.05; **, *P* < 0.01.


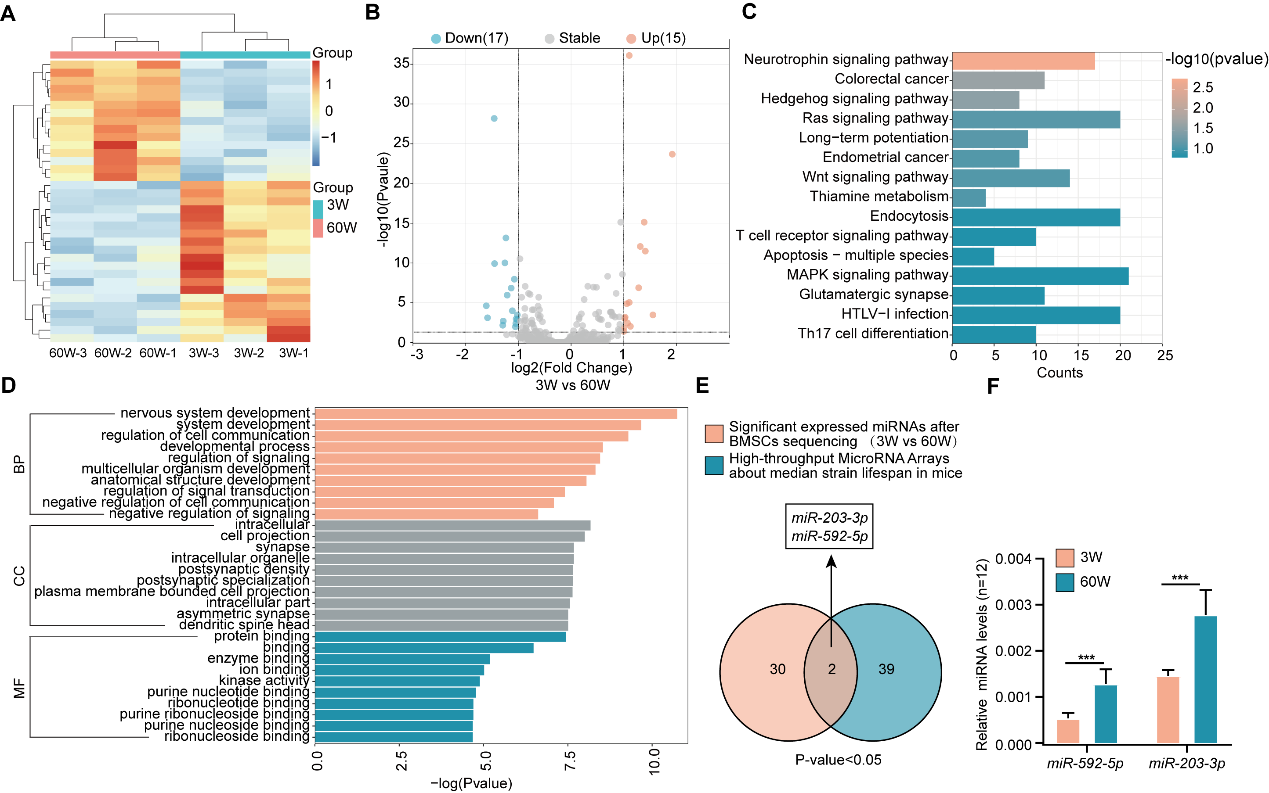


**FigureS2. miRNA sequencing revealed differential expression of miRNAs between 3W-BMSC and 60W-BMSC**

**(A)** Heatmap of differential miRNA expression between the two groups of cells. **(B)** DEGs (|log2 fold change|≥1 and P<0.01) for the two groups of cells in the volcano plot obtained from DESeq2 analysis. **(C)** KEGG pathway analysis of DEGs. **(D)** GO pathway analysis of DEGs. **(E)** Differential miRNA Venn diagram with intersection of miRNA sequencing results for the two groups of cells and high-throughput miRNA array results for the median life span of the mouse species, P-value < 0.05 **(F)** Relative expression of miRNA. ***, *P* < 0.001.


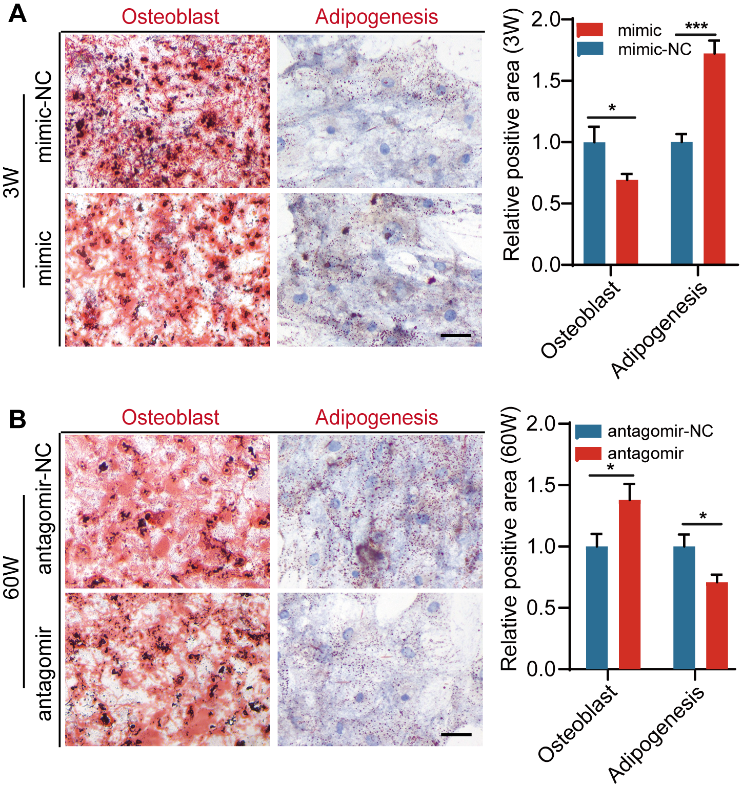


**FigureS3. Effects of miR-203-3p on osteogenic and lipogenic differentiation of BMSCs**

**(A)** Both osteogenic differentiation and adipogenic differentiation in mimic-NC and mimic 3W-BMSCs, determined by Alizarin red staining and Oil red staining with quantification of osteogenic and adipogenic efficiency, Bar: 200 μm. **(B)** Both osteogenic differentiation and adipogenic differentiation in antagomir-NC and antagomir 60W-BMSCs, determined by Alizarin red staining and Oil red staining with quantification of osteogenic and adipogenic efficiency, Bar: 200 μm. *, *P* < 0.05; ***, *P* < 0.001.


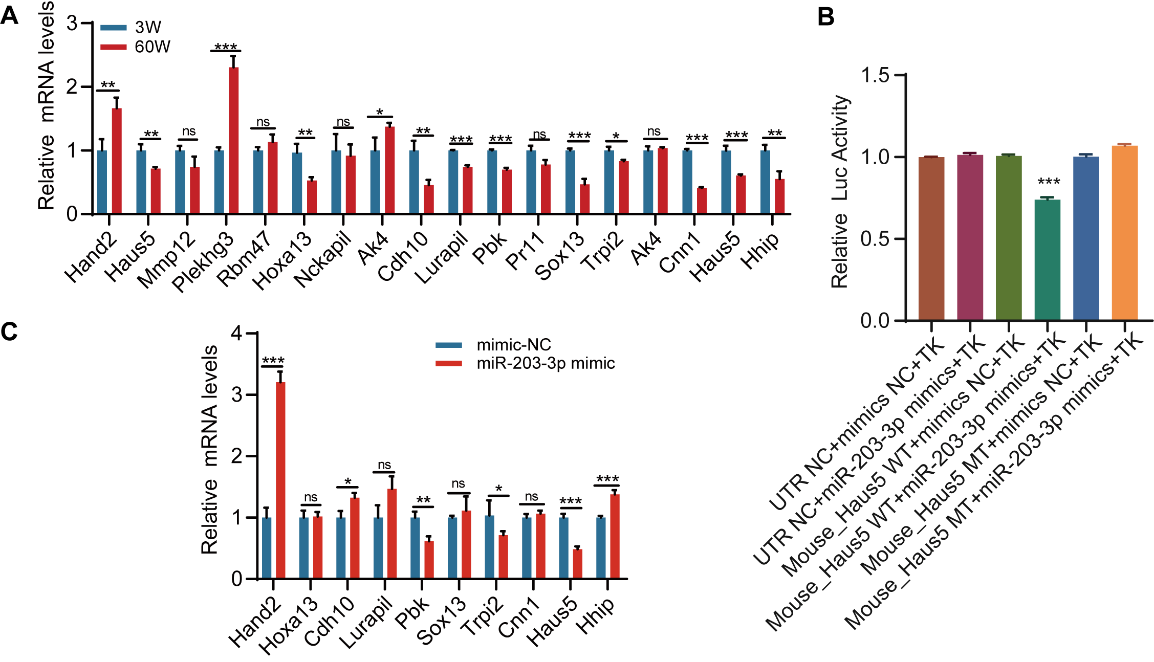


**FigureS4. Screening of miR-203-3p target genes**

**(A)** The relative mRNA expression levels of 18 predicted target genes in 3W and 60W BMSCs **(B)** The relative expression levels of 10 predicted target genes in the control group and miR-203-3p mimic group. **(C)** Dual luciferase reporter gene analysis indicates that miR-203-3p can bind to the 3′-UTR of of Haus5. *, *P* < 0.05; **, *P* < 0.01.


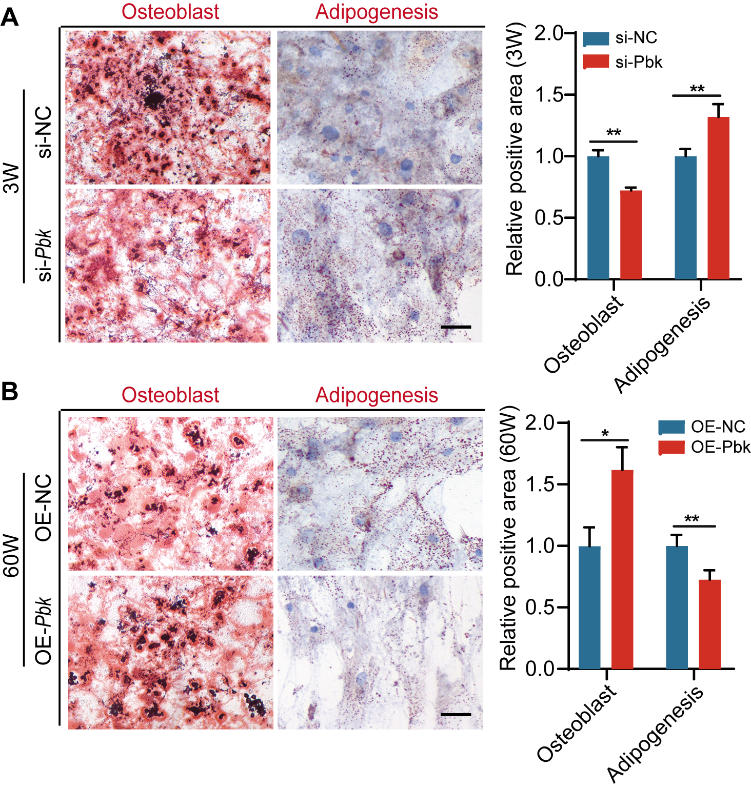


**FigureS5. Effect of Pbk on osteogenic and lipogenic differentiation of BMSCs**

**(A)** Both osteogenic differentiation and adipogenic differentiation in si-NC and si-*Pbk* 3W-BMSCs, determined by Alizarin red staining and Oil red staining with quantification of osteogenic and adipogenic efficiency, Bar: 200 μm. **(B)** Both osteogenic differentiation and adipogenic differentiation in OE-NC and OE-*Pbk* 60W-BMSCs, determined by Alizarin red staining and Oil red staining with quantification of osteogenic and adipogenic efficiency, Bar: 200 μm. *, *P* < 0.05; **, *P* < 0.01.


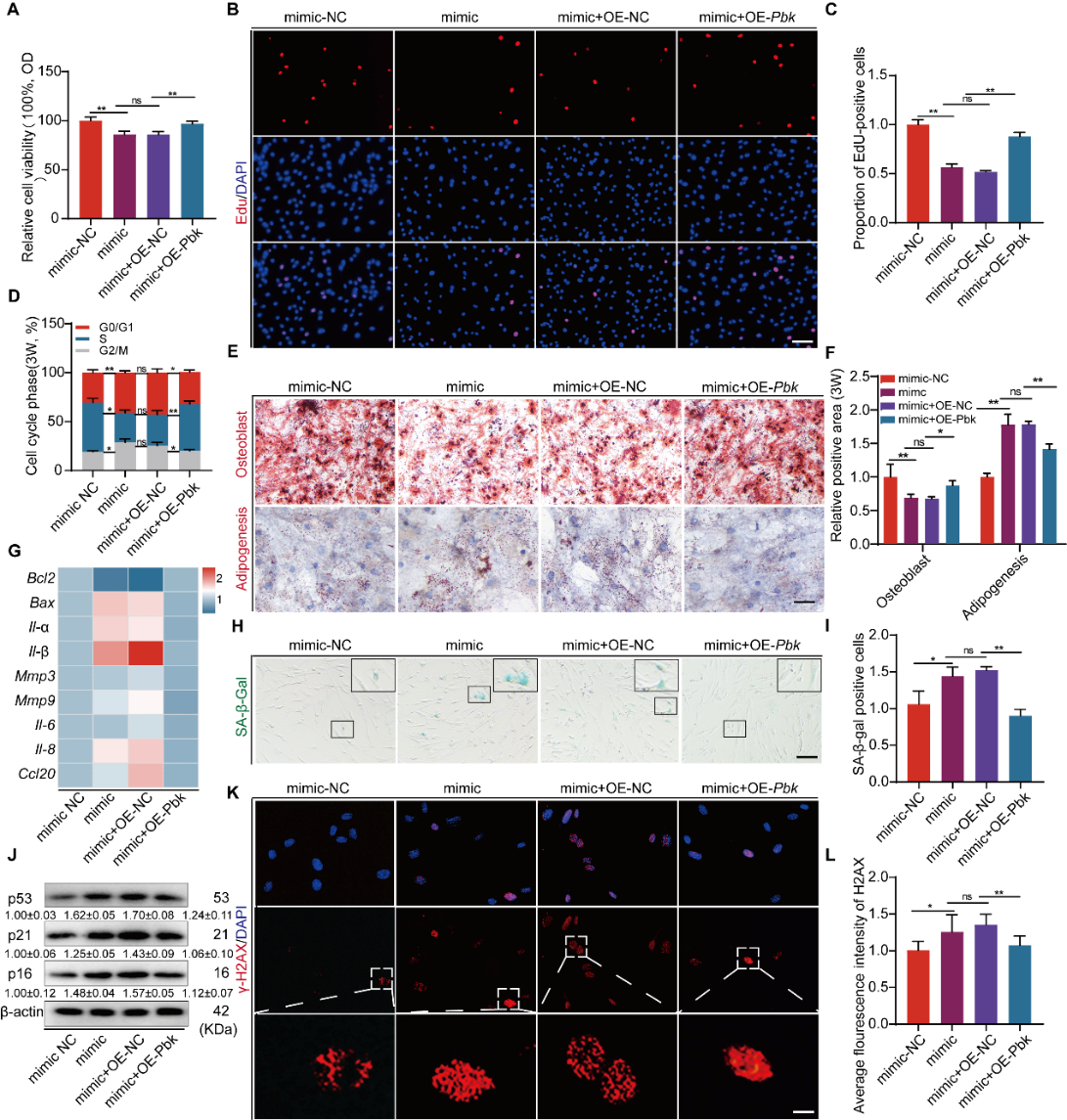


**FigureS6.** **miR-203-3p promotes the senescence of BMSC by downregulating Pbk**

Overexpression of miR-203-3p was performed in young BMSCs and further overexpression of Pbk in the miR-203-3p mimic group to compare the respective phenotypes of the four cell groups. **(A)** CCK8 assay was performed to detect cell viability and counted. **(B-C)** Edu assay to detect cell proliferation and quantify positive cells, Bar: 100 μm. **(D)** Flow cytometry detection of cell cycle in both groups of cells and quantitative analysis of cycle distribution. **(E-F)** Both osteogenic differentiation and adipogenic differentiation in Groups, determined by Alizarin red staining and Oil red staining with quantification of osteogenic and adipogenic efficiency, Bar: 200 μm. **(G)** Heatmap of mRNA expression of genes related to cellular senescence phenotype analysis. **(H-I)** SA-β-gal assay to detect cellular senescence and quantitative analysis of positive cells, Bar: 100 μm. **(J)** Expression levels and quantitative analysis of p16, p21, p53 protein in both groups of cells. β-Actin was used as a loading control. **(K-L)** γ-H2ax immunofluorescence to detect cellular DNA damage and quantification of fluorescence intensity, Bar: 5 μm. *, *P* < 0.05; **, *P* < 0.01.


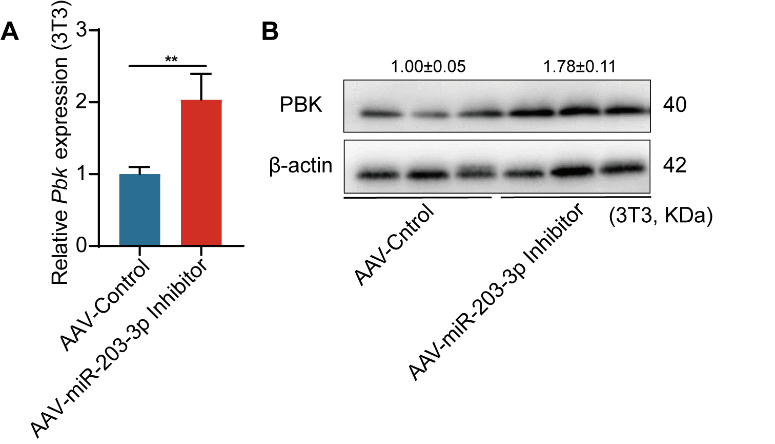


**FigureS7. miR-203-3p Inhibitor adeno-associated virus efficiency verification**

**(A-B)** Detection of mRNA and protein expression levels of Pbk, a classical target gene of miR-203-3p, by RT-qPCR and Western Blot in AAV-control and AAV-miR-203-3p Inhibitor groups. **, *P* < 0.01.


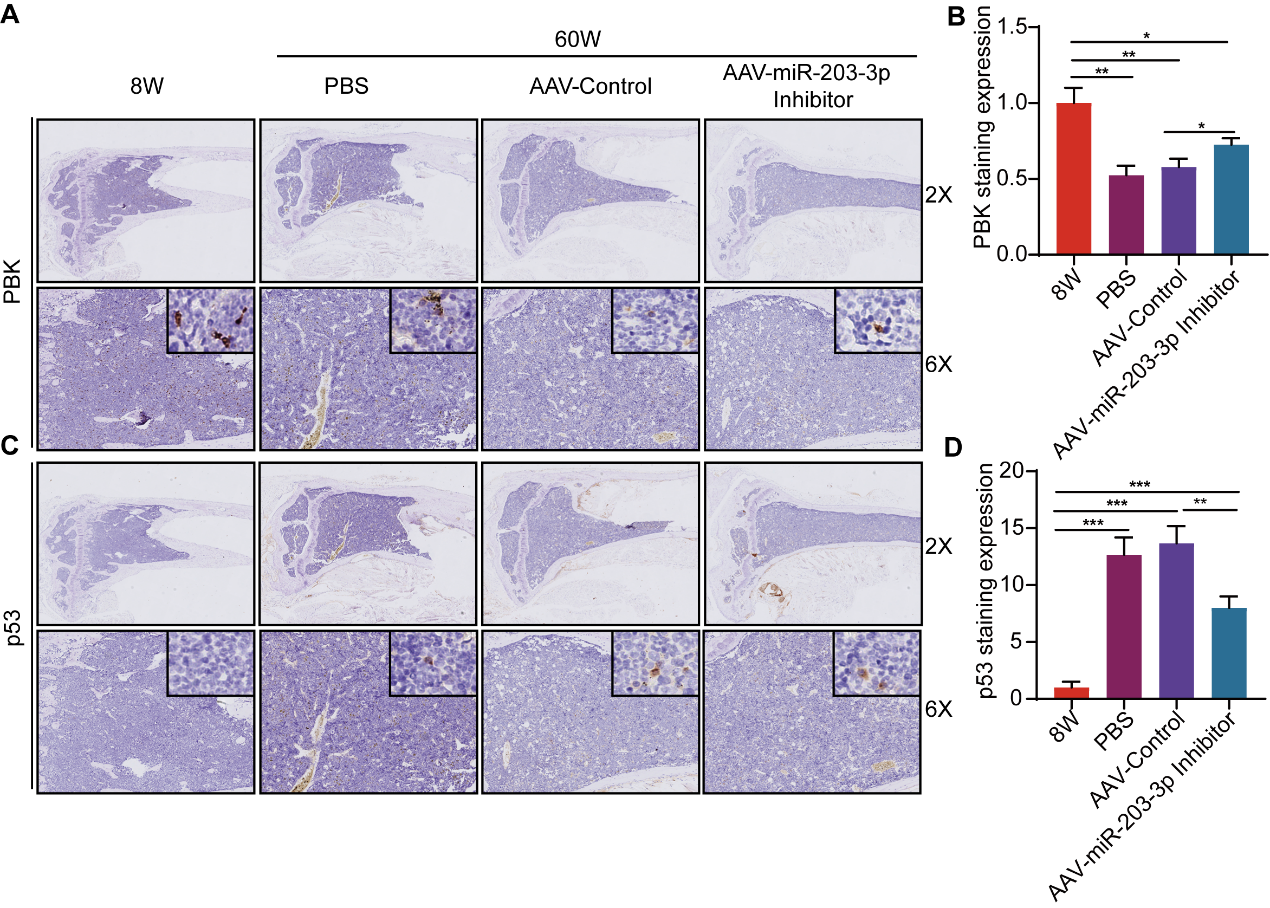


**FigureS8. Expression of Pbk/p53 in the bone marrow cavity of mice**

**(A, B)** Representative Pbk-stained sections of mouse fibula and statistical evaluation of Pbk staining expression. **(C, D)** Representative p53-stained sections of mouse fibula and statistical evaluation of p53 staining expression.

**Supplementary Table 1. Antibodies**

| Antibodies | Source | Identifier |
| --- | --- | --- |
| Rabbit anti-p16 | Abcam | Cat# ab51243 |
| Rabbit anti-p21 | Abcam | Cat# ab109199 |
| Rabbit anti-p53 | Proteintech | Cat# 10442-1-AP |
| Mouse anti-p53 | Abcam | Cat# ab26 |
| Rabbit anti-γ-H2A.X | Cell Signaling Technology | Cat# 4138 |
| Mouse anti-β-Actin | Proteintech | Cat# 66009-1-Ig |
| Rabbit anti-HA tag | Proteintech | Cat# 51064-2-AP |
| Rabbit anti-IgG | Proteintech | Cat# 30000-0-AP |
| Mouse anti-Ubiquitin | Abcam | Cat# PA1-187 |
| Rabbit anti-PBK | Abcam | Cat# ab236872 |

**Supplementary table 2. Primer sequences used in this study.**

| Primer name | Sequence |
| --- | --- |
| *Gapdh* | F: 5’-AACCATGAGAAGTATGACAACAGC-3’ |
|  | R:5’-CATGTGGGGCCATGAGGTCCACCAC-3’ |
| *p16* | F: 5’-GCTCAACTACGGTGCAGATTC-3’ |
|  | R: 5’-GCACGATGTCTTGATGTCCC-3’ |
| *P21* | F: 5’-CCTGGTGATGTCCGACCTG-3’ |
|  | R: 5’-CCATGAGCGCATCGCAATC-3 |
| *P53* | F: 5’-GCGTAAACGCTTCGAGATGTT-3’ |
|  | R: 5’-TTTTTATGGCGGGAAGTAGACTG-3 |
| *Bcl2* | F: 5’-GCTACCGTCGTGACTTCGC-3’ |
|  | R: 5’-CCCCACCGAACTCAAAGAAGG-3’ |
| *Bax* | F: 5’-TGAAGACAGGGGCCTTTTTG-3’ |
|  | R: 5’-AATTCGCCGGAGACACTCG-3’ |
| *Il-1α* | F: 5’-CGAAGACTACAGTTCTGCCATT-3’ |
|  | R: 5’-GACGTTTCAGAGGTTCTCAGAG-3’ |
| *Il-1β* | F: 5’-GAAATGCCACCTTTTGACAGTG-3’ |
|  | R: 5’-TGGATGCTCTCATCAGGACAG-3’ |
| *Mmp3* | F: 5’-ACATGGAGACTTTGTCCCTTTTG-3’ |
|  | R: 5’-TTGGCTGAGTGGTAGAGTCCC-3’ |
| *Mmp9* | F: 5’-CTGGACAGCCAGACACTAAAG-3’ |
|  | R: 5’-CTCGCGGCAAGTCTTCAGAG-3’ |
| *Il-6* | F: 5’-TAGTCCTTCCTACCCCAATTTCC-3’ |
|  | R: 5’-TTGGTCCTTAGCCACTCCTTC-3’ |
| *Il-8* | F: 5’-CAAGGCTGGTCCATGCTCC-3’ |
|  | R: 5’-TGCTATCACTTCCTTTCTGTTGC-3’ |
| *Ccl20* | F: 5’-GCCTCTCGTACATACAGACGC-3’ |
|  | R: 5’-CCAGTTCTGCTTTGGATCAGC-3’ |
| *ND-1* | F: 5’-CTAGCAGAAACAAACCGGGC-3’ |
|  | R: 5’-CCGGCTGCGTATTCTACGTT-3’ |
| *β-globin* | F: 5’-GAAGCGCTTCTAGGGAGCAG-3’ |
|  | R: 5’-GGAGCAGCGATTCTGAGTAGA-3’ |
| U6 | F: 5’-GCTTCGGCAGCACATATACTAAAAT-3’ |
|  | R: 3’-CGCTTCACGAATTTGCGTGTCAT-5’ |
| mmu-miR-203-3p | F: 5’-TGCACTCGAGTACAGCAAAATTAAATACATAAATA-3’ |
|  | R:3’-ATTAGCGGCCGCCTCTTACAGGTTGGACTTTATAGAA-5’ |
| MmiRQP1076 | F: 5’-ATTGTGTCAATATGCGATGATGTAAA-3’ |
| MmiRQP0305 | F:5’-GTGAAATGTTTAGGACCACTAGAAAA-3’ |

**Supplementary material 1: Dual luciferase reporter assay**

Construction of Mouse Pbk 3'UTR (mmu-miR-203-3p) WT vector

Carrier information：PGL3-CMV-LUC-MCS

**
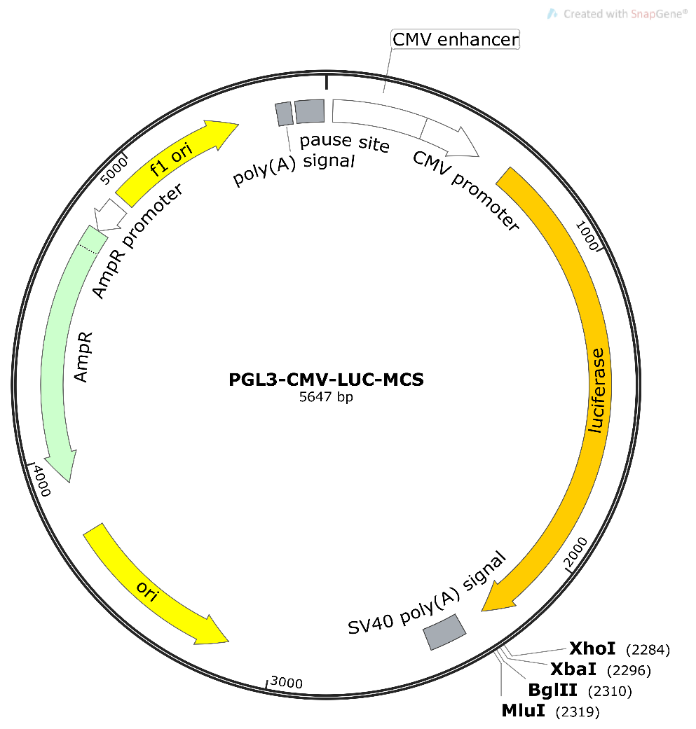
**

| primer： |
| --- |
| 47415FW-60659: AAGATCGCCGTGTGACTCGAGCTTGTATGGGAACTGTTAACTAGATATATG |
| 47415RW-60660: CGCCCCGACTCTAGCACGCGTGCCTGGTACTAAAACTGTCTTTTATTG |

Plasmid name：PGL3-CMV-LUC-Mouse_Pbk 3'UTR (mmu-miR-203-3p) WT


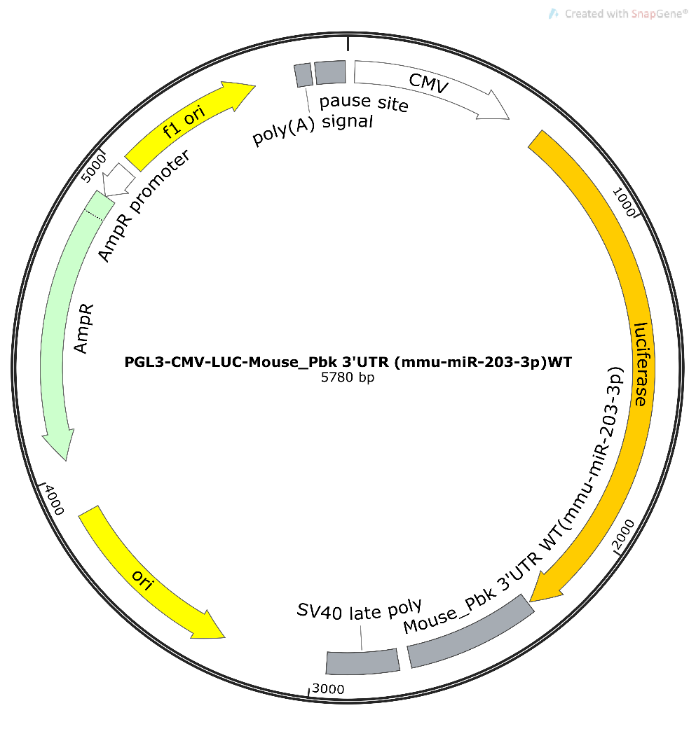


Construction sequence:

CTTGTATGGGAACTGTTAACTAGATATATGTAGTTAATATAACTTATGGTAGCTAGATTCTAGAAGTAGCTTTAACACTAGTGACCCCTGTCTAAGATGACTTAAGAATCAAGGGACCATTGCTTTGTTACAGATCTTTTTAGATATTCTTGCTTCTTTAGTGGGTTACTAAAAATTTCACTACGTACATGTGGTACAGATATCTGTCTGCTCATAGTGTCAGTCCTTCAGCTGGCCTGTCAGCCCATGCGCCCTGGGACTTGAGAAGAGTTCATAAACGTAGCTCCTAGGGTGTCTTGCCTCTCTACACTTAGCTTCTAATTTATTACTTTGTTTCTACTGATTGTGTCTTAAGTCTTTTAAAATAAATGTAAGAATAAACAATAAAAGACAGTTTTAGTACCAGGC

Sequencing analysis:

Primer name：Luc-F

Primer sequence：GAGATCGTGGATTACGTCGC

Sequencing analysis：0030_31121121405282_(47415GI)_[Luc-F]


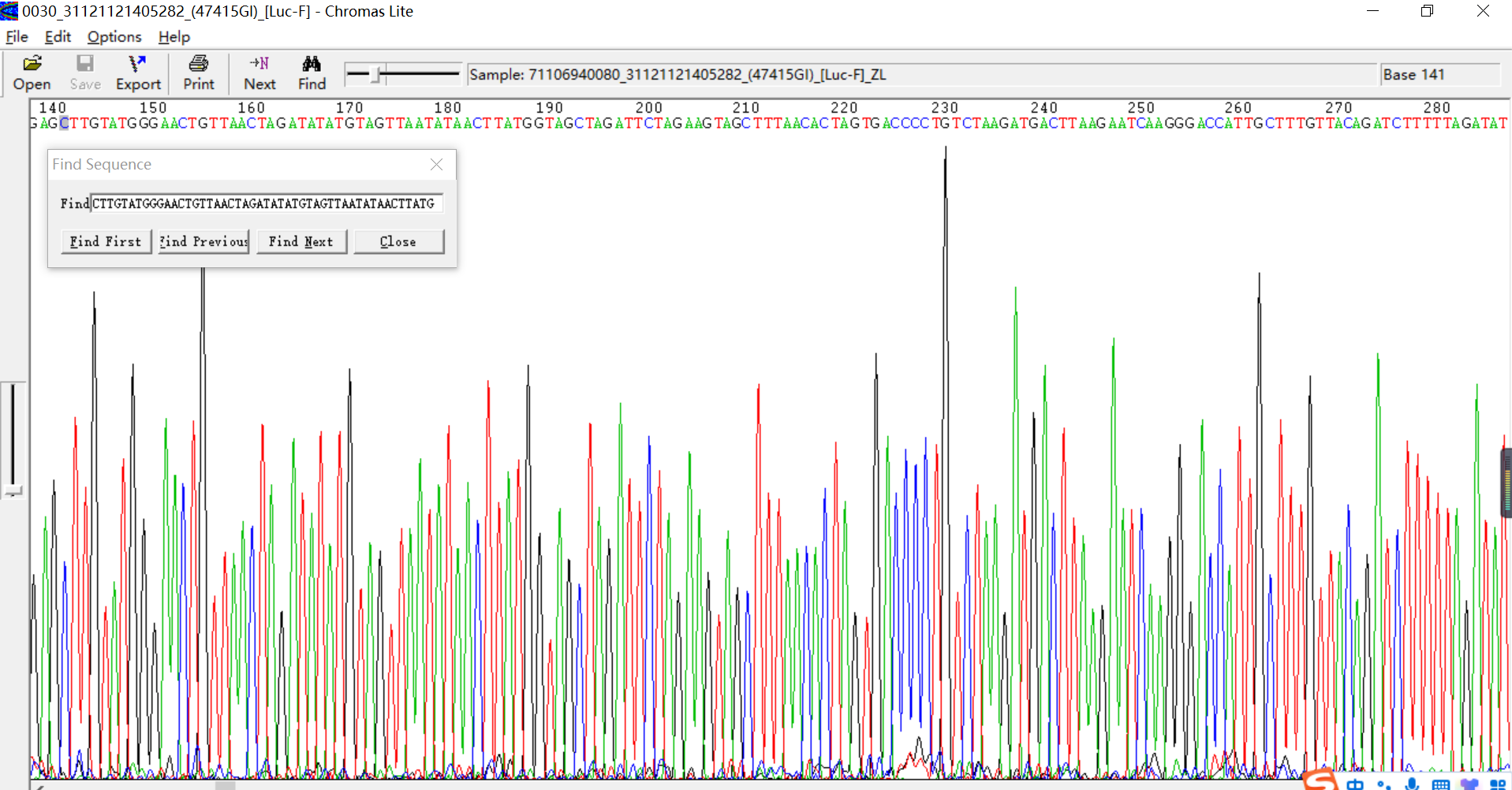


After comparison, the sequence of the inserted fragment in the recombinant clone was completely consistent with that of the target fragment, so the vector was successfully constructed.

Construction of Mouse Pbk 3'UTR (mmu-miR-203-3p) MT vector

Carrier information：PGL3-CMV-LUC-MCS


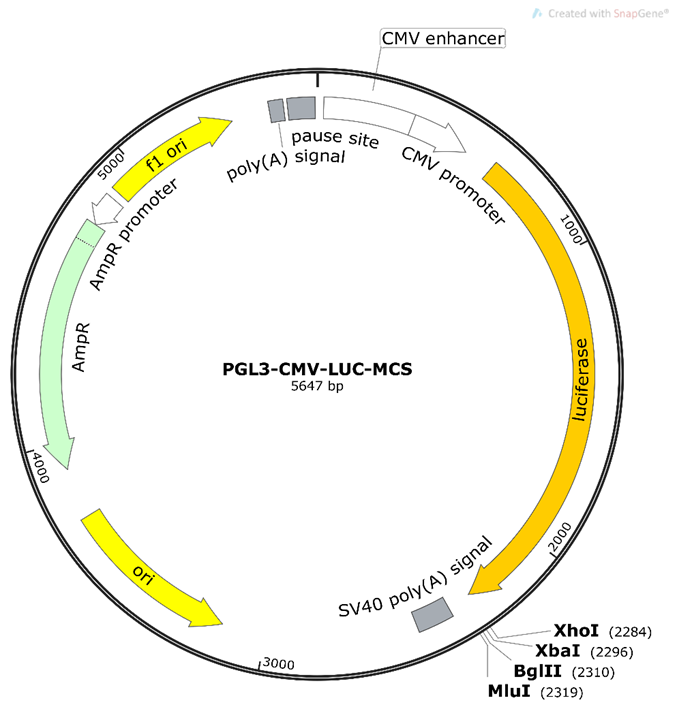


| primer： |
| --- |
| 47415FW-60659: AAGATCGCCGTGTGACTCGAGCTTGTATGGGAACTGTTAACTAGATATATG |
| 47415RW-60660: CGCCCCGACTCTAGCACGCGTGCCTGGTACTAAAACTGTCTTTTATTG |

Plasmid name：PGL3-CMV-LUC-Mouse_Pbk 3'UTR (mmu-miR-203-3p) MT


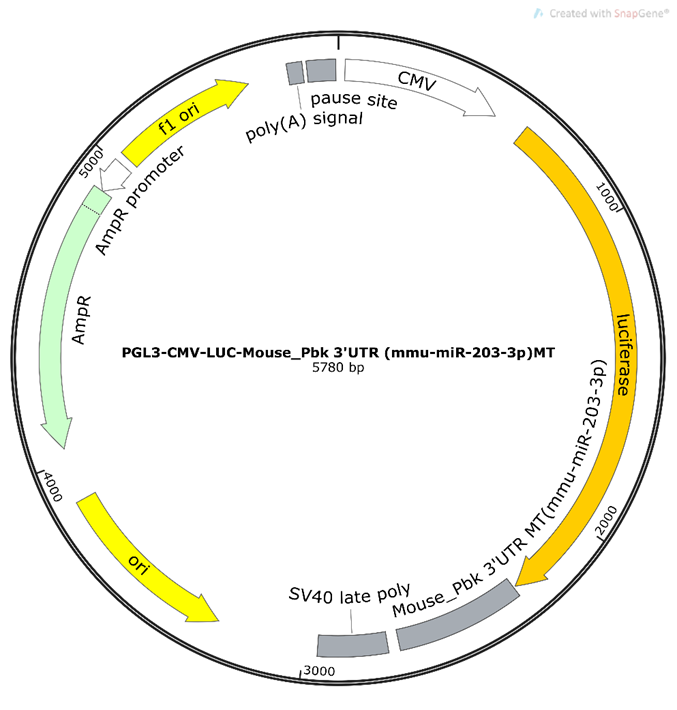


Construction sequence:

CTTGTATGGGAACTGTTAACTAGATATATGTAGTTAATATAACTTATGGTAGCTAGATTCTAGAAGTAGCTTTAACACTAGTGACCCCTGTCTAAGATGACTTAAGAATCAAGGGACCATTGCTTTGTTACAGATCTTTTTAGATATTCTTGCTTCTTTAGTGGGTTA**AGCCCCCGGGAC**CTACGTACATGTGGTACAGATATCTGTCTGCTCATAGTGTCAGTCCTTCAGCTGGCCTGTCAGCCCATGCGCCCTGGGACTTGAGAAGAGTTCATAAACGTAGCTCCTAGGGTGTCTTGCCTCTCTACACTTAGCTTCTAATTTATTACTTTGTTTCTACTGATTGTGTCTTAAGTCTTTTAAAATAAATGTAAGAATAAACAATAAAAGACAGTTTTAGTACCAGGC

(The mutation sequence is shown in the bold line).

Sequencing analysis:

Primer name：Luc-F

Primer sequence：GAGATCGTGGATTACGTCGC

Sequencing analysis：0031_31121121405283_(47417GK)_[Luc-F]


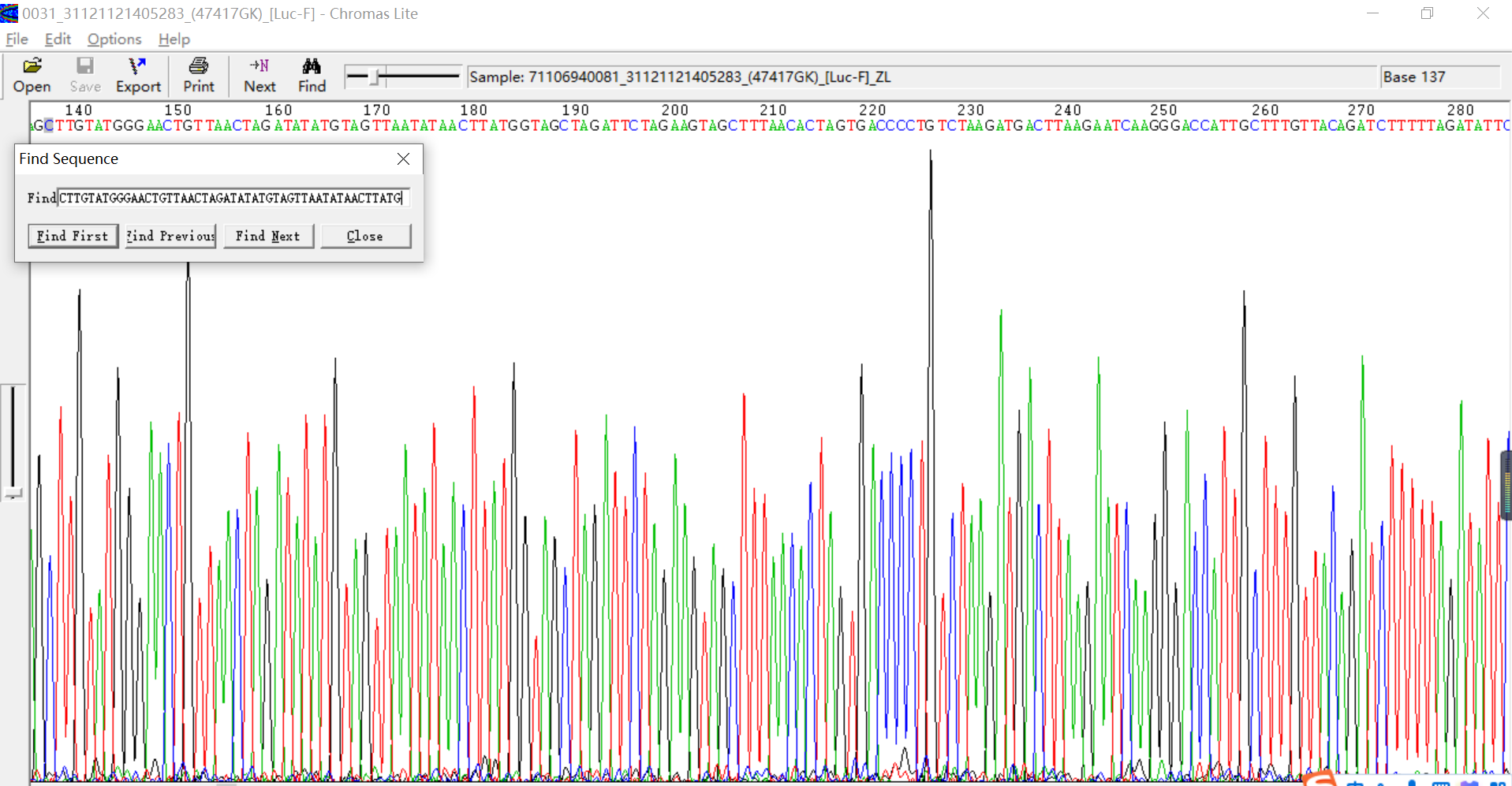


After comparison, the sequence of the inserted fragment in the recombinant clone was completely consistent with that of the target fragment, so the vector was successfully constructed.

**Supplementary material 2: mmu-miR-203-3p Inhibitor adeno-associated virus**

Construction of mmu-miR-203-3p Inhibitor vector

Lentivirus vector information: PGMAAV-10261


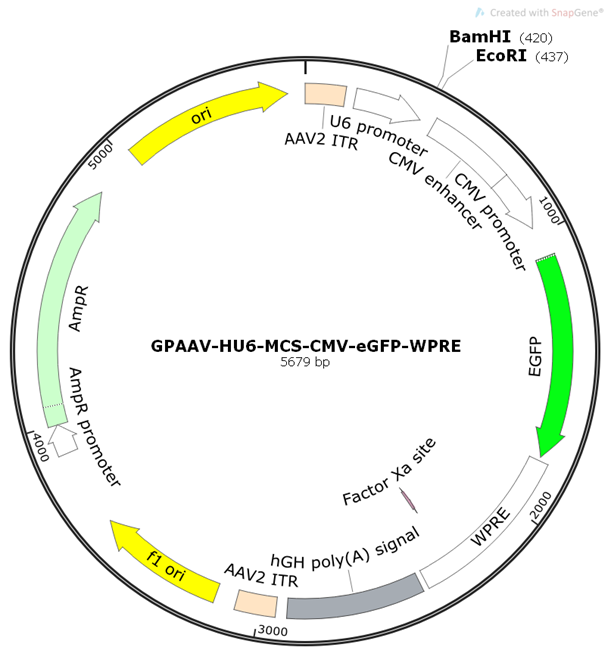


Design of miRNA-Inhibitor primer

| oligo | 5’to 3’ |
| --- | --- |
| Primer-F | GATCCGACGGCGCTAGGATCATCAACCTAGTGGTCCTAATCTAACATTTCACCAAGTATTCTGGTCACAGAATACAACCTAGTGGTCCTAATCTAACATTTCACCAAGATGATCCTAGCGCCGTCTTTTTTG |
| Primer-R | AATTCAAAAAAGACGGCGCTAGGATCATCTTGGTGAAATGTTAGATTAGGACCACTAGGTTGTATTCTGTGACCAGAATACTTGGTGAAATGTTAGATTAGGACCACTAGGTTGATGATCCTAGCGCCGTCG |

Sequencing results of miRNA-Inhibitor vector

| Sequence analysis | mmu-miR-203-3p Inhibitor(PGMAAV-10261) |
| --- | --- |
| GGGGGTCGTTGGGCGGTCAGCCAGGCGGGCCATTTACCGTAAGTTATGTAACGCGGAACTCCCAAGCCTCGAGCTAGAATTCAAAAAAGACGGCGCTAGGATCATCTTGGTGAAATGTTAGATTAGGACCACTAGGTTGTATTCTGTGACCAGAATACTTGGTGAAATGTTAGATTAGGACCACTAGGTTGATGATCCTAGCGCCGTCGGATCCTCGTCCTTTCCACAAGATATATAAAGCCAAGAAATCGAAATACTTTCAAGTTACGGTAAGCATATGATAGTCCATTTTAAAACATAATTTTAAAACTGCAAACTACCCAAGAAATTATTACTTTCTACGTCACGTATTTTGTACTAATATCTTTGTGTTTACAGTCAAATTAATTCTAATTATCTCTCTAACAGCCTTGTATCGTATATGCAAATATGAAGGAATCATGGGAAATAGGCCCTCTTCCTGCCCAGATCATCGATATCGCGTGCGGCCGCAGGAACCCCTAGTGATGGAGTTGGCCACTCCCTCTCTGCGCGCTCGCTCGCTCACTGAGGCCGGGCGACCAAAGGTCGCCCGACGCCCGGGCGGCCTCAGTGAGCGAGCGAGCGCGCAGCTGCCTGCAGGACATGTGAGCAAAAGGCCAGCAAAAGGCCAGGAACCGTAAAAAGGCCGCGTTGCTGGCGTTTTTCCATAGGCTCCGCCCCCCCTGACGAGCATCACAAAATCGACGCTCAAGTCAGAGGTGGCGAAACCCGACAGGACTATAAAGATACCAGGCGTTTCCCCCTGGAAGCTCCCTCGTGCGCTCTCCTGTTCGGACCCTGCCGCTTACCGGATACCTGTCCGCCTTTCTCCCTTCGGGAAGCGTGGCGCTTTCTCATAACTCACGCTGTAGGTATCTCAGTTCGGGGTAGGCCGTTCGCTCCAAGCTGAGCTGGGGGCACGTACCCCCCGGTACAAGCCGACCGCTTGCGCCTAATCCGGACACTTTGGTCCTTGCGTCCCACCCGGATAGAACACCGATTTATCGCCATCGGGAATAATCACTTGCAAACGTGTATTAGCATAGAGGAGGATGATGGAGGGCCTACA | |
| Sequencing primer | CMV-R_R：GGGAACATACGTCATTATTG |
| Sequence analysis | 51682GL-1-primer-CMV-R_R_E02 |


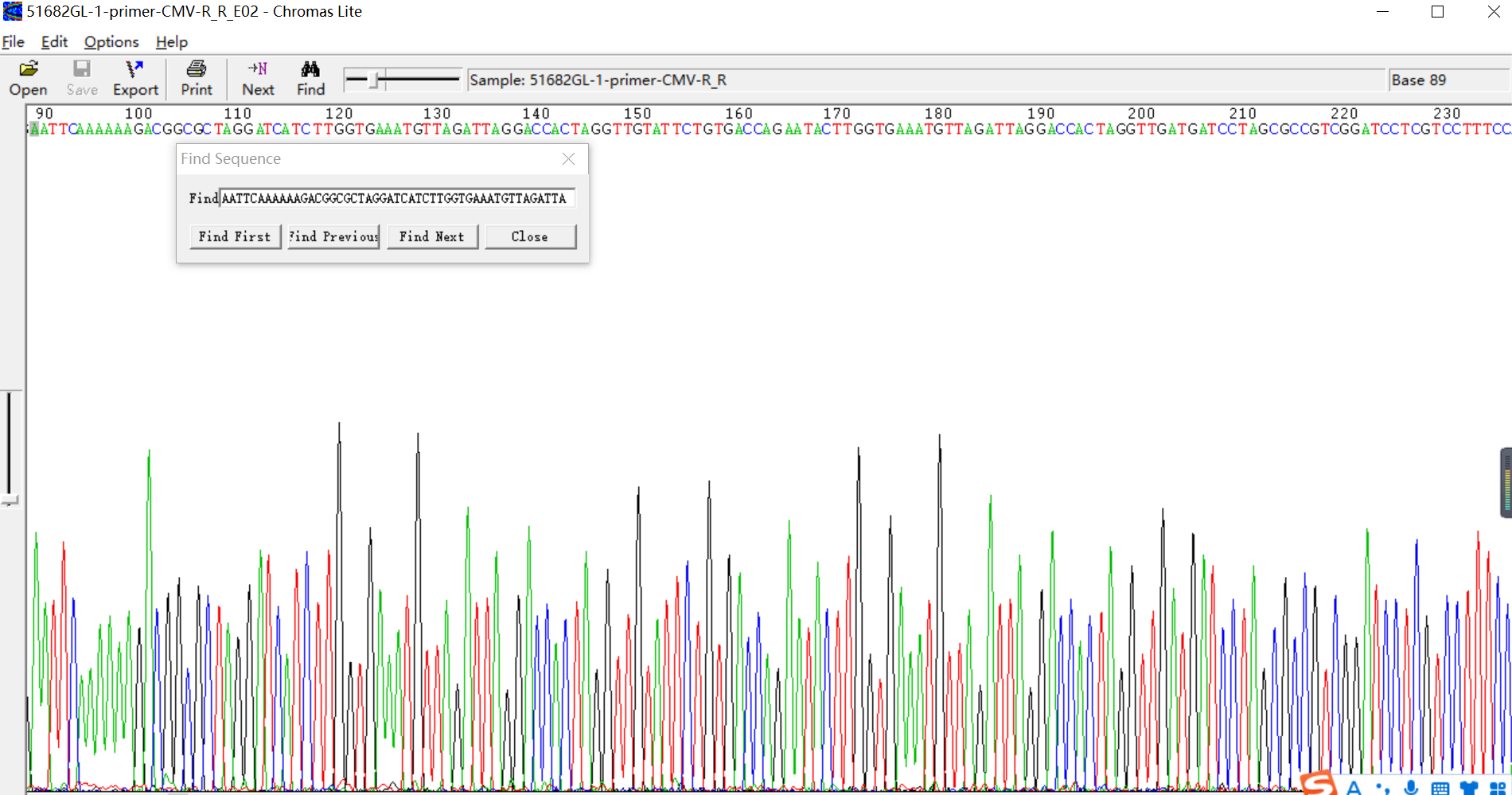


**Supplementary material 3: Packaging of mmu-miR-203-3p Inhibitor adeno-associated virus**

| Sample name | Titer | Dilution ratio | Calculated titer | Mean |
| --- | --- | --- | --- | --- |
| mmu-miR-203-3p Inhibitor(PGMAAV-10261)) -2 | 1.07E+11 | 1.00E+02 | 1.07E+13 | 1.07E+13 |
| mmu-miR-203-3p Inhibitor(PGMAAV-10261)) -4 | 1.11E+09 | 1.00E+04 | 1.11E+13 | 1.09E+13 |
| mmu-miR-203-3p Inhibitor(PGMAAV-10261) -5 | 1.08E+08 | 1.00E+05 | 1.08E+13 | 1.09E+13 |
| mmu-miR-203-3p Inhibitor(PGMAAV-10261)) -6 | 1.16E+07 | 1.00E+06 | 1.16E+13 | 1.11E+13 |
| mmu-miR-203-3p Inhibitor(PGMAAV-10261)) -7 | 1.20E+06 | 1.00E+07 | 1.20E+13 | 1.12E+13 |

The titer of mmu-miR-203-3p Inhibitor(PGMAAV-10261) was 1.12E+13 VG/mL
